# Rewiring faces: advances and outcomes in facial nerve reconstruction after facial vascularized composite allotransplantation

**DOI:** 10.3389/fsurg.2026.1738957

**Published:** 2026-01-30

**Authors:** Leonard Knoedler, Tobias Niederegger, Robert Munzinger, Surbhi Joshi, Thomas Schaschinger, Curtis L. Cetrulo, Christian Festbaum, Andreas Kehrer, Gabriel Hundeshagen, Max Heiland, Steffen Koerdt, Norbert Neckel, Jan O. Voss, Alexandre G. Lellouch

**Affiliations:** 1 Department of Oral and Maxillofacial Surgery, Charité–Universitätsmedizin Berlin, Corporate Member of Freie Universität Berlin and Humboldt-Universität zu Berlin, Berlin, Germany; 2 Division of Plastic and Reconstructive Surgery, Cedars-Sinai Medical Center, Los Angeles, CA, United States; 3 Medical Faculty Heidelberg, University of Heidelberg, Heidelberg, Germany; 4 Department of Plastic, Hand, and Reconstructive Surgery, University Hospital Regensburg, Regensburg, Germany; 5 Department of Hand, Plastic and Reconstructive Surgery, Burn Center, BG Trauma Hospital Ludwigshafen, Department of Plastic and Hand Surgery, University of Heidelberg, Ludwigshafen, Germany; 6 Vascularized Composite Allotransplantation Laboratory, Massachusetts General Hospital, Harvard Medical School, Boston, MA, United States; 7 Innovative Therapies in Haemostasis, INSERM UMR-S 1140, University of Paris, Paris, France

**Keywords:** face transplantation, facial nerve reconstruction, functional facial reanimation, nerve coaptation, vascularized composite allotransplantation

## Abstract

**Background:**

Facial vascularized composite allotransplantation (FVCA) provides transformative restoration for patients with severe craniofacial defects, but successful outcomes depend heavily on facial nerve (FN) reconstruction and reinnervation. Unlike standard nerve repair, FN coaptation in FVCA must address donor–recipient mismatch and immunologic variability. This systematic review synthesizes clinical and preclinical evidence on FN reconstruction strategies in FVCA and their functional outcomes.

**Methods:**

This review adhered to PRISMA 2020 guidelines and was registered with PROSPERO (ID: CRD420251029430). A comprehensive search of PubMed, EMBASE, Cochrane Library, Web of Science, and Google Scholar. Methodological quality was assessed using the Newcastle-Ottawa Scale (NOS) and SYRCLE tool for preclinical studies.

**Results:**

Overall, *n* = 45 (11%) studies [*n* = 41 (91%) human, *n* = 4 (9%) preclinical] published between 2006 and 2025 were included. Human studies were predominantly case reports *n* = 18 (44%), case series *n* = 11 (27%), and cadaveric investigations *n* = 9 (22%). Across *n* = 139 (100%) documented nerve repair interventions (NRIs), direct coaptation was performed in *n* = 20 (14%), most commonly at the FN trunk or its buccal, zygomatic, marginal mandibular, and frontal branches *n* = 28 (20%). Nerve grafting was more frequent, in *n* = 62 (45%), typically using great auricular or thoracodorsal donor nerves; only *n* = 2 (1.4%) NRIs employed dual-level trunk and branch coaptation. Synkinesis was reported in *n* = 11 (7.9%) NRIs, and patient-reported outcomes, though inconsistently collected, indicated improvements in oral continence, speech, social integration, and psychosocial well-being. Secondary revisions occurred in *n* = 27 (19%) and infectious complications in *n* = 12 (8.6%) NRIs. Preclinical rodent and porcine models corroborated clinical evidence that combined motor and sensory nerve repair enhances functional recovery.

**Conclusion:**

FN reconstruction in FVCA is feasible and often results in partial functional recovery. However, outcomes remain heterogeneous and are influenced by surgical approach, immunologic status, and rehabilitative support. Standardized assessment tools should be more widely adopted to improve comparability and guide individualized treatment planning. Translational research and multicenter data collection are needed. FN reconstruction represents both a clinical challenge and an opportunity to improve long-term quality of life in FVCA recipients.

**Systematic Review Registration**: identifier CRD420251029430.

## Graphical Abstract

**Figure:**
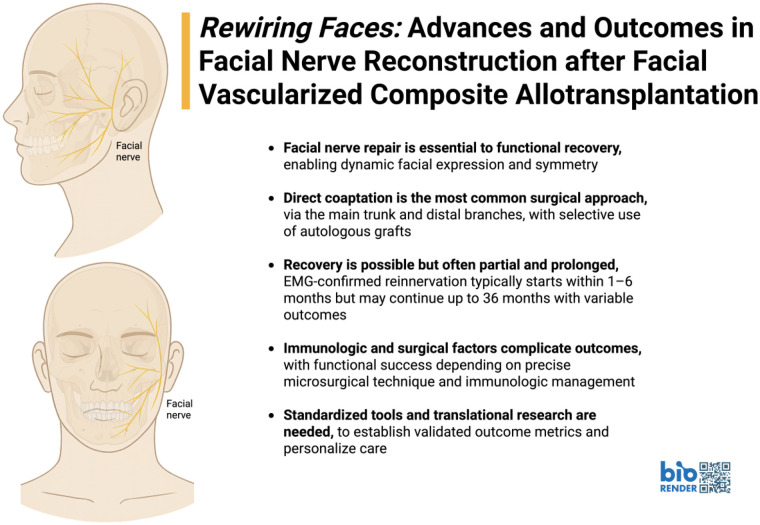

## 1 Introduction

Facial vascularized composite allografts (FVCA), encompassing both full and partial facial transplants, represents an advanced reconstructive technique for selected patients (1–6). Central to the success of this complex surgery is the intricate reconstruction of the facial nerve (FN), which is essential for reanimating facial musculature and restoring expressions (7).

The FNs complex anatomy and critical role in facial movements present unique challenges in the context of transplantation, necessitating a thorough understanding of both microsurgical techniques and neurophysiological principles. Historically, various strategies have been employed to address FN injuries, ranging from direct nerve repair to the use of nerve grafts and conduits (8). Recent advancements have introduced innovative techniques such as cross-FN grafts and the use of motor nerve transfers (9–15).

In the context of facial transplantation, these approaches must be tailored to each patient's unique presentation, such as the specific pattern of nerve involvement, extent of injury (unilateral or bilateral), and individual anatomical or functional considerations, which can make achieving optimal outcomes more challenging (16–19). Factors such as the initial trauma, the timing of FN repair, the distance of FN regeneration, the type of FN coaptation, and the potential for synkinesis or aberrant reinnervation might further complicate the recovery process (20–23). Moreover, the immunological aspects of facial transplantation (high risk of acute rejection, immunogenic skin/mucosa tissue, immunosuppressants) may influence FN regeneration and functional outcomes, adding another layer of complexity to patient management (24, 25).

Given the critical importance of FN reconstruction for the success of facial transplantation, a comprehensive review of current techniques, outcomes, and emerging strategies is warranted. To date, there is a paucity of research synthesizing the current evidence of FN reconstruction in FVCA cases. Therefore, this review aims to consolidate existing literature on FN reconstruction within the context of FVCA, identify knowledge gaps, and provide insights that may guide future research and clinical practice. Because full functional restoration relies on both motor and sensory reinnervation, this review also includes sensory nerve interventions when they form an integral component of the reconstructive strategy.

## 2 Methods

This systematic review followed the PRISMA 2020 guidelines. Due to anticipated variability in study methodologies and reported outcomes, a narrative synthesis was employed in place of a meta-analysis. The complete review protocol was *a priori* registered with PROSPERO (ID: CRD420251029430).

### 2.1 Systematic search and data synthesis

**Figure 1:**
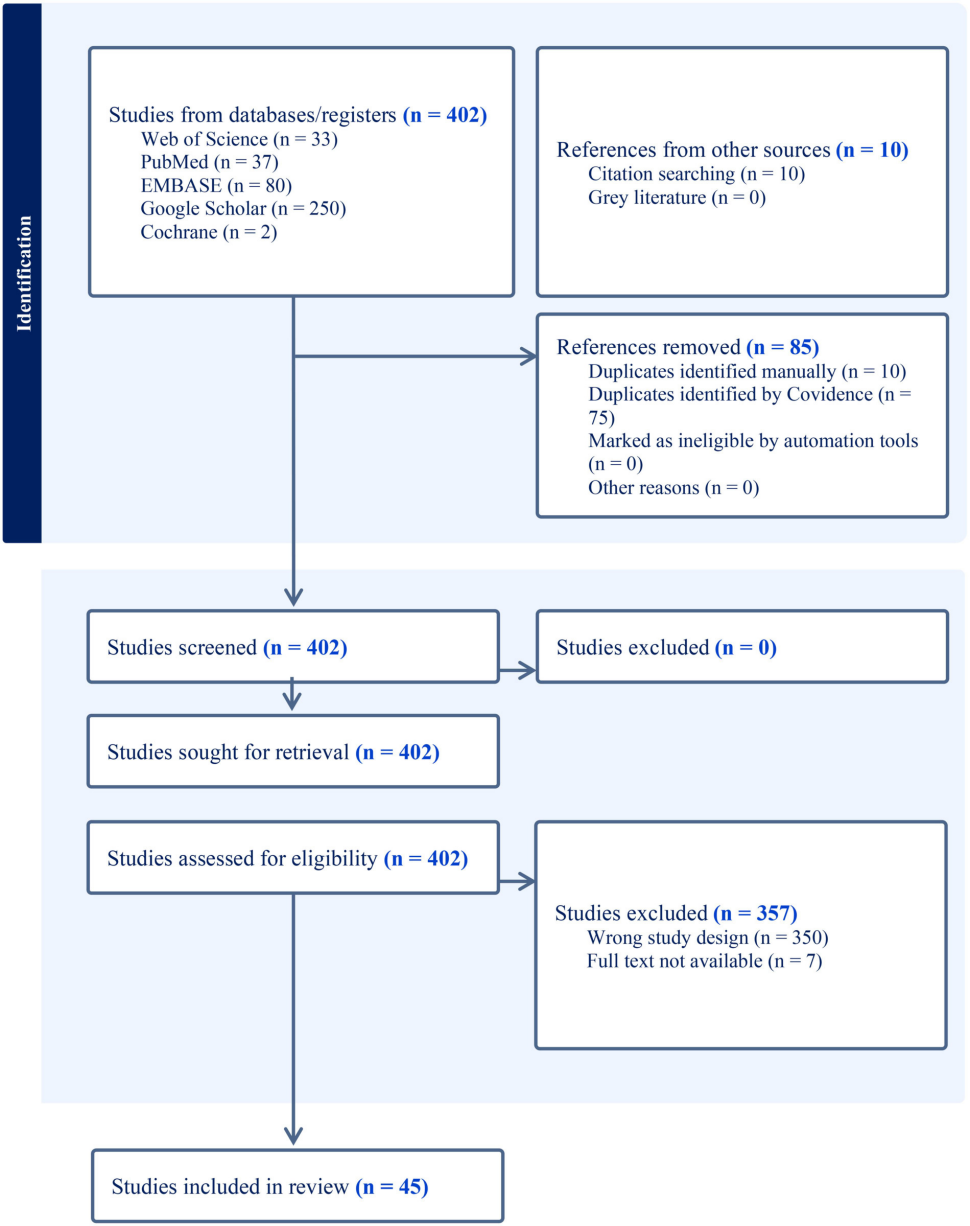
PRISMA 2020 flowchart highlighting the study selection process.

A thorough literature search was conducted across PubMed/MEDLINE, EMBASE, Cochrane Library, Web of Science, and Google Scholar (first 25 pages) to identify all relevant studies published up to July 30th, 2025. Because Google Scholar prioritizes highly cited and field-relevant studies in early pages, screening was limited to the first 25 pages, beyond which studies relevant to this specific research question rarely appear (26, 27). The search strategy focused on two primary concepts: i) “facial vascularized composite allotransplantation” and ii) “facial nerve reconstruction,” incorporating a range of related synonyms and MeSH terms. These two domains were combined using the Boolean operator “AND.” Complete search strings for each database are available in Supplementary Digital Content 1. Additionally, reference lists of all included articles were reviewed to identify any further eligible studies. Studies were included if they presented original, peer-reviewed data investigating facial vascularized composite allotransplantation with a focus on FN reconstruction. All study types, clinical, animal, cadaveric, or *in vitro*, were eligible, provided they directly addressed this topic. Sensory nerve interventions were also included when they formed an integral component of the reconstructive procedure, as comprehensive facial nerve repair encompasses both motor and sensory reinnervation. Articles had to be available in full text and published in English. Studies were excluded if they were not peer-reviewed, did not contain original data (e.g., systematic reviews or meta-analyses), or were unrelated to FVCA or nerve reconstruction. Titles and abstracts were independently screened by three reviewers (T.N., R.M., S.J.), after which full texts were assessed for eligibility. Any discrepancies were resolved in consultation with a senior reviewer (L.K.). The study selection process is illustrated in the PRISMA 2020 flow diagram shown in Figure 1.

To allow for comprehensive qualitative data synthesis, it was not feasible to categorize the data on a per-patient basis, since several publications in the literature describe different aspects of FN reconstruction following FVCA from the same patient and single patients underwent multiple nerve repair procedures. Furthermore, both clinical transplants and cadaveric studies are represented, with some undergoing multiple, sequential, or staged nerve repair procedures employing different techniques. To address this heterogeneity while still providing clinically relevant findings, each distinct clinical human nerve coaptation event is referred to herein as a “nerve repair intervention (NRI).” This classification encompasses any operative method performed in a clinical setting and allows for a granular analysis of surgical techniques without reducing complex, multi-nerve reconstructions into single “case” summaries. Cadaveric and preclinical coaptations were excluded from this specific metric and are discussed separately in the narrative synthesis. Consequently, for biodemographic variables such as age, only ranges rather than means were reported to minimize skewing. In line with this framework, each of the 139 identified clinical NRIs was treated as an independent event in descriptive summaries of surgical technique and outcome, ensuring a consistent representation of clinical reconstructive strategies.

### 2.2 Quality assessment

The quality of included studies was evaluated using appropriate tools depending on study type. Clinical studies were assessed using the Newcastle-Ottawa Scale (NOS), which rates studies across three domains: cohort selection, group comparability, and outcome assessment, with a maximum of nine stars indicating highest quality (28). The NOS is most commonly applied to observational research, including cohort and case-control studies, making it suitable for the predominantly descriptive clinical designs included in this review. Preclinical studies were evaluated using the SYRCLE Risk of Bias tool (29), which adapts the Cochrane framework for animal research and assesses factors such as allocation bias, blinding, and outcome reporting. The tool encompasses ten specific domains, including sequence generation, baseline characteristics, random housing, blinding of caregivers and outcome assessors, incomplete outcome data, and selective reporting, to provide a structured evaluation of internal validity. To determine levels of evidence, the Oxford Centre for Evidence-Based Medicine (OCEBM) framework was applied, ranking randomized trials and systematic reviews as Level I, and grading preclinical studies based on their translational potential (30). Detailed quality appraisal results are provided in Supplementary Digital Content 2, 3, and 4.

### 2.3 Data extraction

Data extraction was performed using a blinded dual-review process. The following parameters were collected from each study: Digital Object Identifier, study title, first author, study species (human or animal), year of publication, study type, sample size, recipient age at transplant, recipient sex, donor age, donor sex, follow-up duration, cause of injury, type of FVCA, donor nerve type, recipient nerve type, type of nerve graft, coaptation sites, suture type, use of intraoperative neuromonitoring, immunosuppression regimen, episodes of graft rejection including number and treatment, functional recovery (e.g., based on House-Brackmann (HB) Grading System (31), evidence of spontaneous facial movement recovery, time to first facial movement, electromyography (EMG) and nerve conduction study findings, facial symmetry at rest, during smiling, and brow elevation, development and severity of synkinesis, patient-reported outcomes including functional and quality-of-life measures, need for revision surgery including type and reason, occurrence of infections or other complications including treatment required, corticosteroid-related issues such as osteonecrosis, hyperglycemia, or weight gain, requirement for additional facial reanimation procedures such as cross-FN grafts or free muscle transfer, and a one-sentence summary of study findings.

## 3 Results

Across all screened studies (*n* = 402, 100%), a total of *n* = 41 (11%) human studies and *n* = 4 (0.9%) preclinical models met the *a priori* determined inclusion and exclusion criteria. The year of publication ranged from 2006 to 2025. Study types were predominantly case reports (*n* = 18, 44%) and case series (*n* = 11, 27%). Furthermore, *n* = 9 (22%) studies included human cadavers. The mean (SD) NOS-score was 5.0 (0.0), indicating overall low to moderate methodological quality.

### 3.1 Study demographics

Recipient age ranged from 19 to 64 years. Donor age ranged from 18 to 99 years. A total of *n* = 139 (100%) NRIs were reported. The majority of NRIs were performed in males (*n* = 71, 51%), and *n* = 29 (21%) in females. Follow-up durations ranged from 2 months to over 6 years.

Reported causes of facial injury necessitating FVCA and NRI encompassed ballistic trauma, burns, animal attacks, Neurofibromatosis type 1, and oncologic resections. The extend of FVCA ranged from partial face grafts involving perioral, nasal, or zygomatic units to full-face transplants, including osteomyocutaneous components. Full study demographics are available in Table 1.

**Table 1:** Patient demographics.

DOI	Study title	First author	Year of publication	Study type	Sample size	Recipient age	Recipient sex	Donor age	Donor sex	Follow-up time	Cause of Injury	Type of facial VCA
DOI: 10.1016/S0140-6736 (06)68935-6	First human face allograft: early report	Devauchelle et al.	2006	Case report	1	38	f	46	f	4 months	Animal Attack	Central and lower face (nose, lips, chin, adjacent cheeks)
DOI: 10.1097/01.sap.0000227486.28556.3e	Technical and Anatomical Considerations of Face Harvest in Face Transplantation	Baccarani et al.	2006	Cadaveric study	2	N/A	N/A	N/A	N/A	N/A	N/A	N/A
DOI: 10.1056/NEJMoa072828	Outcomes 18 months after the first human partial face transplantation.	Dubernard et al.	2007	Case report	1	38	f	46	f	18 months	Dog bite	N/A
DOI: 10.1016/j.bjps.2007.12.014	Osteocutaneous face transplantation	Follmar et al.	2007	Cadaveric study	1	N/A	N/A	N/A	N/A	N/A	N/A	Myocutaneous
DOI: 10.1007/s00104-007-1446-x	Facial allograft transplantation: Fiction or reality? Sques in a fresh human cadaver model	Meßmer et al.	2008	Cadaveric study	4	N/A	N/A	Range: 50–90	f	N/A	N/A	Type 1: Skin flap (sub-SMAS technique); Type 2: Osteocutaneous flap (Le Fort III segment)
DOI: 10.1016/S0140-6736 (08)61276-3	Human facial allotransplantation: a 2-year follow-up study	Guo et al.	2008	Case report	1	30	m	25	m	2 years	Animal Attack	Partial face (nose, upper lip, parotid gland, front wall of maxillary sinus, infraorbital wall, zygomatic bone)
DOI: 10.1097/PRS.0b013e3181882146	Face Transplant Graft Procurement: A Preclinical and Clinical Study	Meningaud et al.	2008	Cadaveric study and case report	1	29	m	N/A	m	N/A	NF	Myocutaneous
DOI: 10.1097/PRS.0b013e3181954e8c	Mini-temporalis transfer as an adjunct procedure for smile restoration	Terzis et al.	2009	Cohort study	31	N/A	N/A	N/A	N/A	More than 3 months	Trauma	N/A
DOI: 10.1016/S0140-6736 (09)61155-7	Near-total human face transplantation for a severely disfigured patient in the USA	Siemionow et al.	2009	Case report	1	45	f	N/A	f	More than 5 months	Ballistic trauma	Osteomyocutaneous
DOI: 10.1001/archfacial.2009.80	The Technical and Anatomical Aspects of the World's First Near-Total Human Face and Maxilla Transplant	Alam et al.	2009	Case report	1	45	f	N/A	f	N/A	N/A	N/A
DOI: 10.1097/prs.0b013e3181c2a5cc	Facial Transplantation: An Anatomic and Surgical Analysis of the Periorbital Functional Unit	Vasilic et al.	2010	Cadaveric study	12	N/A	N/A	Range: 49–99	8 m, 4f	N/A	N/A	Periorbital functional unit (eyelids)
DOI: 10.1097/PRS.0b013e318230c77b	An Update on Facial Transplantation Cases Performed between 2005 and 2010	Siemionow et al.	2010	Case series	13	Range: 28–59	11 m, 2 f	Range: 25–65	11 m, 2f	N/A	Animal attack, neurofibromatosis, ballistic/explosion trauma, burn/electrical injury, cancer	Myocutaneous and osteomyocutaneous VCA
DOI: 10.1016/j.transproceed.2011.06.030	Reconstruction of a severe facial defect by allotransplantation in neurofibromatosis type 1: A case report	Sicilia-Castro et al.	2011	Case report	1	35	m	18	m	6 months	Neurofibromatosis type 1	Lower two-thirds of the face (osteomyocutaneous allograft), including chin osseous segment, skin, subcutaneous tissue, lips, perioral muscles, parotid glands, facial nerves
DOI: 10.1097/SLA.0b013e318226a607	Full Face Transplant The First Case Report	Barret et al.	2011	Case report	1	30	m	41	m	More than 4 months	Ballistic trauma	Osteomyocutaneous VCA
DOI: 10.1111/j.1600-6143.2010.03406.x	Feasibility, Reproducibility, Risks and Benefits of Face Transplantation: A Prospective Study of Outcomes	Lantieria et al.	2011	Case series	5	Range: 27–39	m	N/A	m	Range: 7–38 months	Ballistic trauma, burn injury, neurofibromatosis	Osteomyocutaneous and myocutaneous VCA
DOI: 10.1111/j.1600-6143.2010.03368.x	Restoration of Facial Form and Function After Severe Disfigurement from Burn Injury by a Composite Facial Allograft	Pomohac et al.	2011	Case report	1	59	m	60	m	15 months	Electrical burn	Osteomyocutaneous
DOI: 10.1097/PRS.0b013e31825dc25c	Novel surgical technique for full face transplantation	Pomahac et al.	2012	Case series	3	N/A	N/A	N/A	N/A	N/A	Severe facial defects	Full face allotransplantation (including scalp, eyelids, nose, maxilla, muscles, nerves, and vessels)
DOI: 10.1097/prs.0b013e31828bd394	Nerve transfers for facial transplantation: a cadaveric study for motor and sensory restoration.	Audolfsson et al.	2013	Cadaveric study	15	N/A	N/A	Mean: 68.2, range: 49–92	10 m, 5f	N/A	N/A	Simulated face transplantation (midface, upper/lower lip, buccal region)
DOI: 10.1177/000348941312201106	Long-term outcomes of facial nerve function in irradiated and nonirradiated nerve grafts	Leong et al.	2013	Cohort study	42	Mean: 53.2, range: 16–80	24 m, 18 f	N/A	N/A	2 years	Facial fractures, neuroma	N/A
DOI: 10.1097/PRS.0b013e31828bd394	Facial allotransplantation procurement using a transparotid approach: A new anatomical model	Horta et al.	2014	Cadaveric study	3	N/A	N/A	N/A	N/A	N/A	N/A	Full face allotransplant (including fronto-temporo-parietal scalp, eyelids, nose, muscles, lips, nerves, vessels)
DOI: 10.1002/micr.22216	A functional periorbital subunit allograft: Vascular, anatomic, and technical considerations for future subunit facial transplantation	Mathes et al.	2014	Cadaveric study	12	N/A	N/A	N/A	N/A	N/A	N/A	Periorbital subunit (eyelids, medial/lateral canthal areas, inferior/superior periorbital skin)
DOI: 10.1016/j.bjps.2014.05.046	Eyelid Transplantation: Lessons from a Total Face Transplant and the Importance of Blink	Sosin et al.	2015	Case report	1	37	m	N/A	N/A	1,1 years	Trauma (avulsive ballistic injury)	Total face, double-jaw, tongue
DOI: 10.1097/prs.0000000000000798	Long-Term Multifunctional Outcome and Risks of Face Vascularized Composite Allotransplantation	Roche et al.	2015	Case report	1	55	m	N/A	N/A	3 years	Ballistic injury	N/A
DOI: 10.1097/SCS.0000000000002110	Referred facial sensation on the hand after full face transplantation	Uysal et al.	2016	Case report	1	19	m	37	N/A	N/A	Burn injury	Full face transplant (excluding eyelids)
DOI: 10.1212/WNL.0000000000002409	Surgical Optimization of Motor Recovery in Face Transplantation	Aycart et al.	2016	Case series	2	P1: 25, P2:57	P1:m, P2:w	N/A	N/A	3,5 years	P1: electrical burn; P2: animal attack	Full face allotransplantation
DOI: 10.1097/SCS.0000000000002305	The First Immediate Face Transplant in the World	Maciejewski et al.	2016	Case report	1	31	m	30	m	2 years	Traumatic injury	Osteomyocutaneous
DOI: 10.1097/SLA.0000000000001597	Facial nerve regeneration after facial allotransplantation: A longitudinal clinical and electromyographic follow-up of lip movements during speech	De et al.	2017	Case report	1	54	m	-	-	3 years	Ballistic trauma	Lower two-thirds of face, maxillae, nasal bones, mandible, hard palate
DOI: 10.1016/j.bjps.2017.02.025	The Effects of Lip-Closure Exercise on Lip Strength and Function Following Full Facial Transplantation: A Case Report	Bridget et al.	2017	Case report	1	40	f	N/A	N/A	1 year	Burn injury	Osteomyocutaneous VCA
DOI: 10.1044/2017_AJSLP-16-0101	Assessment of Emotional Expressions after Full-Face Transplantation	Topçu et al.	2017	Case series	3	P1:28, P2:37, P3:22	m	N/A	N/A	Up to 2 years	Burn injury	Osteomyocutaneous VCA
DOI: 10.1155/2017/8789724	Recovery of facial expressions using functional electrical stimulation after full-face transplantation.	Topçu et al.	2018	Case series	2	N/A	N/A	N/A	N/A	N/A	N/A	Total face VCA
DOI: 10.1186/s12984-018-0356-0	Image-based Analysis of Emotional Facial Expressions in Full Face Transplants	Bedeloglu et al.	2018	Case series	2	P1:22, P2:39	m	N/A	N/A	Up to 4 years	N/A	Total face VCA (with and without eyelids)
DOI: 10.1007/s10916-018-0895-8	Software-based video analysis of functional outcomes of face transplantation	Fischer et al.	2018	Case series	7	N/A	N/A	N/A	N/A	1 year	Burn injury, ballistic trauma, acid attack	Face VCA
DOI: 10.1002/micr.30360	The Helsinki approach to face transplantation	Lindford et al.	2019	Case series	2	P1:35, P2:58	m	N/A	N/A	30,5 months	N/A	Osteomyocutaneous VCA
DOI: 10.1016/j.bjps.2018.08.030	Recognizing Emotional Expression as an Outcome Measure After Face Transplant.	Dorante et al.	2020	Case series	6	42	N/A	N/A	N/A	2	N/A	Face VCA
DOI: 10.1001/jamanetworkopen.2019.19247	Full facial retransplantation in a female patient—Technical, immunologic, and clinical considerations	Kauke et al.	2021	Case report	1	52 (age at retransplantation), first transplant at age 45	f	Second donor: 36, first donor: 56	f	0,5 years	Lye burn	Full-face re-/transplant
DOI: 10.1111/ajt.16696	Neuromotor Speech Recovery Across Different Behavioral Speech Modifications in Individuals Following Facial Transplantation	Eshghi et al.	2021	Cohort study	7	Range: 28–61	5 m, 2f	N/A	N/A	Early group: 2 months, late group: 42 months	Trauma, burn injury, cancer	Full or partial facial allografts (including lips, facial muscles, ± osteomyocutaneous components)
DOI: 10.3389/fneur.2020.593153	Face Transplant: Current Update and First Canadian Experience	Govshievich et al.	2021	Case report	1	64	m			18 months	Ballistic trauma	Le Fort III and bilateral sagittal split osteotomies in addition to skin
DOI: 10.1097/PRS.0000000000007890	Facial Nerve Revascularization Strategies in Facial Restoration	Khajuria et al.	2022	Case series	5	41	N/A	N/A	N/A	6 years	N/A	Face VCA
DOI: 10.1097/GOX.0000000000004038	Re-cognizing the new self: The neurocognitive plasticity of self-processing following facial transplantation	Azevedo et al.	2022	Case report	1	25	m	N/A	N/A	2 years	Ballistic trauma	Osteomyocutaneous
DOI: 10.1073/pnas.2211966120	Facial Expression after Face Transplant: An International Face Transplant Cohort Comparison	Dorante et al.	2023	Cohort study	13	Mean: 40 ± 14	11 m, 2f	Mean: 40 ± 13	N/A	Mean: 3.6 ± 2.2	Burn injury, ballistic trauma	Full and partial face VCA, including midface, lower two-thirds, scalp and nasal structures
DOI: 10.1097/PRS.0000000000010242	Anatomical study of trigeminal-facial nerve communications: Application to facial transplant surgery	Iwai et al.	2025	Cadaveric study	6	N/A	N/A	Mean: 76,5	3 m, 3f	N/A	N/A	Face VCA

DOI, digital object identifier; VCA, vascularized composite allotransplantation; N/A, not available/not applicable; m, male; f, female; PX, patient X; SMAS, superficial musculoaponeurotic system; Le Fort III, anatomical classification of midface fracture used for orientation in facial surgery.

### 3.2 Facial nerve repair approaches

Reconstruction of the FN was a central component of NRI in all reported FVCA procedures. In *n* = 20 (14%) NRIs, direct coaptation of the donor FN branches to recipient FN stumps was performed. The most common coaptation sites (20%, *n* = 28) involved the main FN trunk and its distal branches—primarily the buccal, zygomatic, marginal mandibular, and frontal branches. Bilateral coaptation was reported in *n* = 6 (4.3%) NRIs, while *n* = 2 (1.4%) involved selective unilateral repair.

Nerve grafting was more common than direct coaptation, occurring in *n* = 62 (45%) of NRIs. Both autologous and allogenic grafts, most frequently the great auricular and thoracodorsal nerves, were used to bridge motor nerve gaps, particularly in complex or revision surgeries. While detailed outcome comparisons are limited, early data suggest that functional recovery (e.g., facial movement and symmetry) was achievable in both grafted and non-grafted NRIs, with no consistent evidence of inferior outcomes associated with graft use. Notably, in *n* = 2 (1.4%) NRIs dual-level coaptation at both the proximal trunk and distal branches of the FN was reported, reflecting a more aggressive reconstructive strategy aimed at optimizing reinnervation. However, whether this approach offered superior motor recovery remains unclear due to the absence of comparative or longitudinal outcomes.

Microsurgical suture techniques most commonly involved 8–0 to 10–0 nylon sutures, used in 32% (*n* = 45) NRIs, while alternative methods included fibrin-based adhesives such as fibrin sealant (*n* = 1,.07%) and Tisseel® (i.e., a commercially available fibrin glue used to promote hemostasis and tissue adhesion in microsurgery) in *n* = 5 (3.5%) NRIs. However, there is currently not enough comparative evidence within the reviewed NRIs indicating that the use of Tisseel® led to different functional outcomes compared to standard nylon sutures. In selected NRIs (*n* = 3, 2.2%), intraoperative neuromonitoring was used. Overall, FN repair was consistently prioritized in surgical planning, underscoring its critical role in achieving motor reanimation and facial symmetry. Further information is provided in Table 2.

**Table 2:** Nerve reconstruction details.

Study title	First author	Year of publication	Study type	Donor nerve	Recipient nerve	Type of nerve graft	Coaptation sites	Suture type	Intraoperative neuromonitoring used?	Immunosuppressive regimen	Graft rejection episodes	Functional and sensory recovery	Spontaneous facial movement recovery	Time to first movement	EMG/NCS findings	Facial symmetry	Synkinesis development	Patient reported outcomes
Technical and Anatomical Considerations of Face Harvest in Face Transplantation	Baccaraniet al.	2006	Cadaveric study	FN, mental, orbital	FN, mental, orbital	N/A (theorized coaptation only)	N/A	N/A	N/A	N/A	N/A	N/A	N/A	N/A	N/A	N/A	N/A	N/A
Osteocutaneous face transplantation	Follmar et al.	2007	Cadaveric study	FN, trigeminal, greater auricular, supraorbital, infraorbital and mental nerves	FN, trigeminal, greater auricular, supraorbital, infraorbital and mental nerves	N/A	N/A	N/A	N/A	N/A	N/A	N/A	N/A	N/A	N/A	N/A	N/A	N/A
Facial allograft transplantation: Fiction or reality?: Sques in a fresh human cadaver model	Meßmer et al.	2008	Cadaveric study	Type 1: Trigeminal nerve (sensory); Type 2: FN (motor)	N/A	N/A	Type 1: Trigeminal nerve branches (sensory); Type 2: FN (motor)	N/A	N/A	N/A	N/A	N/A	N/A	N/A	N/A	N/A	N/A	N/A
Facial Transplantation: An Anatomic and Surgical Analysis of the Periorbital Functional Unit	Vasilic et al.	2010	Cadaveric study	FN (temporal, zygomatic, buccal branches)	N/A	N/A	N/A	N/A	N/A	N/A	N/A	N/A	N/A	N/A	N/A	N/A	N/A	N/A
Nerve transfers for facial transplantation: a cadaveric study for motor and sensory restoration.	Audolfsson et al.	2013	Cadaveric study	Sensory: Cervical plexus branches (greater auricular, supraclavicular, transverse cervical, lesser occipital nerves) Motor: Masseter nerve	Sensory: Mental nerve, infraorbital nerve; Motor: Buccal branches of FN	Direct coaptation (nerve transfer; no graft used)	Cervical plexus branches → mental nerve. Masseter nerve → buccal branches of the FN. Supraorbital nerve → infraorbital nerve (simulated)	N/A	N/A	N/A	N/A	N/A	N/A	N/A	N/A	N/A	N/A	N/A
Facial allotransplantation procurement using a transparotid approach: A new anatomical model	Horta et al.	2014	Cadaveric study	FN (main trunk, temporofacial/cervicofacial divisions & individual branches)	N/A	None (technique avoids nerve grafts)	Not specified (theoretical coaptation at temporofacial/cervicofacial divisions or distal branches)	N/A	N/A	N/A	N/A	N/A	N/A	N/A	N/A	N/A	N/A	N/A
A functional periorbital subunit allograft: Vascular, anatomic, and technical considerations for future subunit facial transplantation	Mathes et al.	2014	Cadaveric study	Motor: Zygomatic and buccal branches of the FN; Sensory: Infraorbital, supraorbital, and supratrochlear nerves	N/A	N/A	FN branches (zygomatic, buccal	N/A	N/A	N/A	N/A	N/A	N/A	N/A	N/A	N/A	N/A	N/A
Anatomical study of trigeminal-facial nerve communications: Application to facial transplant surgery	Iwai et al.	2025	Cadaveric study	Trigeminal nerve branches [ophthalmic (V1), maxillary (V2), mandibular (V3)] and facial nerve (FN) branches	N/A	N/A	V3 (mandibular nerve): Auriculotemporal (6/6 cases) and buccal (6/6 cases) nerves communicated with FN Mental nerve communicated in 3/6 cases; V2 (maxillary nerve): Infraorbital nerve communicated with FN in 3/6 cases; V1 (ophthalmic nerve): No communication with FN	N/A	N/A	N/A	N/A	N/A	N/A	N/A	N/A	N/A	N/A	N/A
Face Transplant Graft Procurement: A Preclinical and Clinical Study	Meningaud et al.	2008	Cadaveric study and case report	FN & trigeminal	FN & trigeminal	Direct coaptation	Trunk	N/A	N/A	N/A	N/A	N/A	N/A	N/A	N/A	N/A	N/A	N/A
First human face allograft: early report	Devauchelle et al.	2006	Case report	FN branches (zygomatic, buccal, mandibular), infraorbital, mental nerves	FN stump (mandibular branch on left), infraorbital, and mental nerves	None (direct coaptation)	Left mandibular branch of FN, b/l infraorbital and mental nerves	10/0 Prolene (arteries), 9/0 Prolene (veins/nerves)	N/A	ATG, TAC, MMF, PDN; donor bone marrow infusions	1 episode at day 20; grade IN/AII rejection; treated with steroids	Not found (sensation recovery at 14 weeks; partial motor recovery at 12 weeks)	Partial (upper lip movement at 12 weeks; incomplete smile)	3 months (12 weeks)	Not specified (sensation assessed via Semmes-Weinstein testing)	Slight lower lip sagging; otherwise, good integration	N/A	Positive psychological acceptance; return to social life
Outcomes 18 months after the first human partial face transplantation.	Dubernard et al.	2007	Case report	N/A	N/A	N/A	N/A	N/A	N/A	Intravenous ATG (TMG, Genzyme) for 10 days, oral TAC (target trough levels, 10 to 15 ng per milliliter throughout the first month), MMF (2 g per day), PDN (250 mg on day 1, 100 mg on day 2, and 60 mg per day through day 12, followed by a gradual taper)	Extracorporeal photochemotherapy was introduced at 10 months to prevent recurrence of rejection	N/A	N/A	N/A	N/A	N/A	N/A	N/A
Human facial allotransplantation: a 2-year follow-up study	Guo et al.	2008	Case report	FN branches (details not fully specified)	FN Stump (right buccal branches; anastomosis attempted)	Direct coaptation (no nerve grafts used)	FN anastomosis (exact branches unspecified)	N/A	N/A	TAC, MMF, STR, humanized IL-2 receptor MAB	3 episodes at 3, 5, 17 months; treated with TAC dose adjustment/steroid pulses	Partial motor recovery (incomplete smile), sensory recovery at 3 months (Semmes-Weinstein testing)	Partial (upper lip movement; incomplete smile)	N/A	N/A	Slight lower lip sagging; improved appearance post-revision	N/A	Positive psychological acceptance; reintegration into society
Near-total human face transplantation for a severely disfigured patient in the USA	Siemionow et al.	2009	Case report	FN, vagus, hypoglossal	FN, vagus, hypoglossal	B/l FN connected with standard epineural repair, donor vagus nerve used for interpositional graft & attached with 2 upper division trunks of right FN; left hypoglossal interpositional graft attached to upper division trunk of recipient FN. Both grafts were connected to main trunk of donor nerve	Donor (main trunk), recipient (upper division of FN)	N/A	N/A	Induction: Rabbit ATG, MPDN; Maintenance: TAC, MMF, low dose PDN	Day 47-Graft mucosa (Tx with steroid bolus)	Slow but progressing, as shown by improved facial mimetics with symmetric smiling and upper lip occlusion, upper lip and lower eyelid movements were imperfect	N/A	N/A	N/A	N/A	N/A	5 months PO, rates self-appearance 8/10, optimistic about rebuilding social life
The Technical and Anatomical Aspects of the World's First Near-Total Human Face and Maxilla Transplant	Alam et al.	2009	Case report	N/A	N/A	N/A	N/A	N/A	N/A	N/A	N/A	N/A	N/A	N/A	N/A	N/A	N/A	N/A
Reconstruction of a severe facial defect by allotransplantation in neurofibromatosis type 1: A case report	Sicilia-Castro et al.	2011	Case report	FN, infraorbital nerves, mental nerves (branches of trigeminal nerve)	FN stumps (remaining after tumor resection), infraorbital & mental nerves	Allograft	FN, infraorbital & mental nerves (specific branches not detailed)	Fibrin sealant used adjunctively, no further information was available	N/A	Induction: Basiliximab (20 mg), TAC (6 mg), MPDN; Maintenance: PDN (10 mg/d), MMF (1.5 g/d), TAC (target 8–10 ng/mL).	1, POD 28 (Banff grade III); TAC dose adjustment was taken into account, MPDN pulse therapy, topical TAC were used	Qualitative descriptions only: motor recovery began at 6 months in levator labii and buccinator muscles; sensory recovery at 3–6 months	Yes	6 months (levator labii and buccinator)	Reinnervation evidence noted (electroneuromyographic examination confirmed motor recovery) Latency/amplitude changes were not quantified	N/A	N/A	High satisfaction: improved speech, oral feeding, and social reintegration (no standardized scales like FACE-Q used)
Full Face Transplant The First Case Report	Barret et al.	2011	Case report	Trigeminal (supraorbital, infraorbital, mandibular nerves) & buccal, zygomatic, orbicularis oculi, frontal branches of FN	Trigeminal (supraorbital, infraorbital, mandibular nerves) & buccal, zygomatic, orbicularis oculi, frontal branches of FN	Direct coaptation	N/A (end-to-end anastomosis)	N/A	N/A	Induction: TMG, PDN; TAC, MMF (switched to sirolimus)	Yes (2, from MMF switched to Sirolimus)	At 4 months PO: regained active movement of the frontalis muscles, lateral portion of zygomatic muscles, upper orbicularis oculi muscles & unrestricted masticatory movements. Movement in some areas were still partial, pt was unable to close his eyes completely	N/A	N/A (able to start soft diet 2 weeks PO)	EMG 75 days PO: no signs reinnervation; 120 days initial signs of muscle activity detected	N/A	N/A	N/A (immediate PO reaction positive)
Restoration of Facial Form and Function After Severe Disfigurement from Burn Injury by a Composite Facial Allograft	Pomohacet al.	2011	Case report	FN, buccal, infraorbital	FN, buccal, infraorbital	Neurorrhaphy	FN branches (5 on right side, 6 on left side), immediately anterior to parotid gland	N/A	N/A	Induction: MPDN, Rabbit ATG, MMF; Maintenance: MMF & TAC	Yes, at day 17 (Tx: steroids)	Gradual improvement after 6 months	Yes	N/A (by 1 yr PO pt could smile symmetrically & gained control of upper lip)	N/A	1 year PO pt had symmetric smile	No	Pt returned to living facility 5 weeks PO, fully integrated into community with enhanced social capacity, reconnected with divorced wife and daughter & prioritized function over aesthetic appearance
Eyelid Transplantation: Lessons from a Total Face Transplant and the Importance of Blink	Sosin et al.	2015	Case report	FN branches: buccal, zygomatic; supraorbital, supratrochlear nerves preserved but not coapted	FN stump (middle branch required nerve grafting)	nerve grafting used for middle branch due to insufficient length	Zygomatic, buccal, middle branch required nerve grafting	N/A	Yes (nerve stimulation with checkpoint surgical, which is a nerve stimulator)	Not specified (corticosteroids mentioned in discussion)	N/A	7.5 months (13.5 months after FT) after revision Sx to correct involuntary blink reflex; right eye (10–40%), left eye (60–90%); HB-scores not reported; voluntary blink preserved, involuntary blink improved post-transplant (70% right eye, 100% left eye) but temporarily impaired post-revision	Yes (improved involuntary blink post-transplant)	N/A	N/A	Qualitative improvement noted	N/A	Pt reported comfort and artificial tear use noted
Long-Term Multifunctional Outcome and Risks of Face Vascularized Composite Allotransplantation	Roche et al.	2015	Case report	N/A	N/A	N/A	N/A	N/A	N/A	Maintenance therapy consists of corticoids, TAC, MMF in minimal doses	Rejection successfully treated	N/A	Yes	1 month	Yes	N/A	N/A	N/A
Referred facial sensation on the hand after full face transplantation	Uysal et al.	2016	Case report	Infraorbital, supraorbital, mental, and frontal branches of the FN	FN trunk (lower branches)	Direct coaptation (no graft specified)	B/l infraorbital, supraorbital, and mental nerves; Frontal branches coapted separately; Lower FN branches coapted to recipient's FN trunk	N/A	N/A	Induction: ATG, PDN; Maintenance: TAC, MMF, PDN	N/A	Not explicitly reported; EMG at 6 months confirmed reinnervation in frontalis, orbicularis oculi and orbicularis oris muscles	Partial recovery (e.g., eyebrow movement, lip pursing) but emotional expressions (smile, anger) remained incomplete at 2 years	Motor activity detected by EMG at 6 months; functional movements observed gradually thereafter	Reinnervation in frontalis, orbicularis oculi and orbicularis oris muscles at 6 months but incomplete recovery of emotional facial expressions was noted	Aesthetic outcome implied by “adequate recovery of primary sensory modalities”	N/A	Referred facial sensations, touch on hands/fingers perceived as sensations on lips, forehead, and earlobes (topographically mapped), improved sensory modalities (pain, light touch) seen but incomplete two-point discrimination was observed
The First Immediate Face Transplant in the World	Maciejewski et al.	2016	Case report	FN & branches (trunk left side, branches of right side); mental nerve	FN & branches (trunk left side, branches of right side); mental nerve	Direct coaptation of FN; autologous nerve forearm graft for b/l mental nerves	Direct coaptation FN, autologous forearm nerve graft for mental nerves (donor mental nerves connected to recipient auricular magnus using grafts)	N/A	N/A	Induction: ATG, TAC, MMF, MPDN; Maintenance: TAC, MMF, MPDN	Yes (POD 34, grade 2 histopathology, tx with steroids)	N/A (b/l sensation recovery by 8 weeks PO)	Yes	N/A	N/A	N/A	N/A	N/A
Facial nerve regeneration after facial allotransplantation: A longitudinal clinical and electromyographic follow-up of lip movements during speech	De Letter et al.	2017	Case report	Not explicitly reported (assumed FN)	Not explicitly reported (assumed FN stump)	N/A	N/A	N/A	N/A	N/A	Yes (1 episode at 4 months, tx with immunosuppressants)	Not reported; EMG showed reinnervation starting at 1 month, clinical improvement over 38 months	EMG activity detected at 1 month, gradual clinical improvement	1 month (EMG activity); visible movement timing not specified	Early reinnervation at 1 month, increasing amplitude, decreasing reaction times over 38 months	N/A	N/A	Facial Disability Index (FDI), Voice Handicap Index (VHI), Speech Handicap Index (SHI), Oral Health Impact Profile (OHIP-14) scores provided at multiple time points
The Effects of Lip-Closure Exercise on Lip Strength and Function Following Full Facial Transplantation: A Case Report	Bridget al.	2017	Case report	N/A	N/A	Allograft	5 FNs	N/A	N/A	N/A	N/A	Lip strengthening exercise	Ability to drink from a straw & communicate via facial expression	N/A	N/A	Yes	N/A	Better straw use, enhanced facial communication
Full facial retransplantation in a female patient—Technical, immunologic, and clinical considerations	Kauke et al.	2021	Case report	FN branches	FN remnants (from prior transplant	N/A	FN coaptation was performed at the level of divisions, with coaptation of three FNs divisions on the left and four FN divisions on the right (7 coaptation sites)	N/A	N/A	Induction: ATG, MMF, MPDN; Maintenance: PDN (10 mg QD), TAC (3 mg BID, goal 8–10 ng/mL), MMF (1,000 mg BID); Prophylaxis: Valganciclovir (CMV), trimethoprim-sulfamethoxazole (PCP)	1st transplant: chronic antibody-mediated rejection (AMR) and recurrent T cell-mediated rejection (TCMR), leading to irreversible graft loss at 88 months. Retransplant: Grade III TCMR at 3 and 4 months post-retransplant, treated with STR and alemtuzumab	Not reported (clinical improvement noted at 6 months post-retransplant)	N/A	N/A	N/A	N/A	N/A	Psychosocial challenges noted (e.g., pain, functional limitations) but no quantitative scores (e.g., FDI, FACE-Q) were provided
Face Transplant: Current Update and First Canadian Experience.	Govshievich et al.	2021	Case report	N/A	Microsurgical anastomoses of FN (3 branches) & infraorbital nerves were performed bilaterally	Allograft	N/A	N/A	N/A	N/A	N/A	N/A	N/A	N/A	N/A	N/A	N/A	N/A
Re-cognizing the new self: The neurocognitive plasticity of self-processing following facial transplantation	Azevedo et al.	2022	Case report	N/A	N/A	N/A	N/A	N/A	N/A	N/A	N/A	N/A	Yes	N/A	EMG 2 years PO noted improvement, FN function & motor recruitment that correlated with improved speech & facial function	Not reported but based on included photos pt had excellent facial/smile symmetry	N/A	Pt returned to pre-injury daily activities
An Update on Facial Transplantation Cases Performed between 2005 and 2010	Siemionow et al.	2010	Case series	FN & its branches repaired in 8 pts, 4 pts had infraorbital nerve repairs, 2 pts had mental nerve repairs, 1 pt had buccal & supraorbital nerve repair	All direct coaptation of nerves done except for 1st pt in mental nerve repair; donor nerve stumps placed near mental foramen. Most underwent b/l FN repair, 1st pt had left mandibular branch repair but right FN was not well coapted	Direct coaptation (no graft specified)	Not specified (theoretical coaptation at temporofacial/cervicofacial divisions or distal branches)	N/A	N/A	Pt 1: Induction (ATG, MMF, PDN), Maintenance (TAC, sirolimus, MMF, PDN, IL-2R Ab); pt 2: Induction (TAC, MMF,MPDN, IL-2R Ab), Maintenance (TAC, MMF,PDN, IL-2R Ab); pt 3: Induction (Antilymphocyte serum, PDN), Maintenance (TAC, MMF, PDN); pt 4: Induction (Rabbit ATG, MPDN, TAC), Maintenance (TAC, MMF, PDN)	N/A	Qualitative descriptions mentioned only for 4 pts: 1st pt (upper lip motion by 12 weeks, lower lip motion at 4 months, mouth closure by 6 months, smile at 14–18 months, chin & nose pyramidal muscle motion seen by 12 months); 2nd pt (no time frame reported, eat drink and speak normally, FN not fully functional); 3rd pt (at 6 months orbicularis oculi contraction, by 9 months spontaneous mimicry, by 10 days eat and speak, at 12 months facial motor function); 4th pt (no time frame-upper lip occlusion, facial mimicry, eat & drink from cup, speak clearly) qualitative descriptions only: motor recovery began at 6 months in levator labii and buccinator muscles; sensory recovery at 3–6 months	N/A	N/A	N/A	N/A	N/A	N/A
Feasibility, Reproducibility, Risks and Benefits of Face Transplantation: A Prospective Study of Outcomes	Lantieri et al.	2011	Case series	B/l FN, trigeminal	B/l FN, trigeminal	N/A	N/A	All nerve coaptations were glued with Tisseel R (a fibrin sealant)	N/A	Induction: ATG, TAC, MMF; Maintenance: TAC, MMF, PDN	3 pts: 1 episode	Data present of 3 pts (in 2 pts voluntary muscle contraction of zygomatic & orbicular oris; in 1 pt right zygomatic was absent, right orbicularis oris was absent along with absent complete mouth closure	Yes	N/A	EMG: By 6 months PO voluntary contraction of left orbicularis oculi was seen, in both orbicularis oris by 12 months motor & sensory innervarion b/l restored; 11 months PO no motor recovery was observed, at right side-correction of coaptation (appeared macroscopically normal IO)	N/A	N/A	Overall improvement with SF-36, MCS & QOL testing
Novel surgical technique for full face transplantation	Pomahac et al.	2012	Case series	FN branches (zygomatic, buccal), sensory nerves (supraorbital, infraorbital, mental)	FN & its branches, hypoglossal nerve & sensory nerve stumps of supraorbital, infraorbital, mental	Allografts (without parotidgland)	FN branches (zygomatic, buccal), sensory nerves (supraorbital, infraorbital, mental)	N/A	No	Induction immunosuppression	No	N/A	Yes	N/A	N/A	Improved aesthetics reported (due to exclusion of parotid glands), but no scoring done	N/A	N/A
Surgical Optimization of Motor Recovery in Face Transplantation	Aycart et al.	2016	Case series	Pt 1: FN divisions (superior/inferior) and thoracodorsal nerve (autograft). Pt 2: FN branches, masseter nerve (transfer), and great auricular nerve (graft)	Pt 1: superior and inferior divisions of the left FN, right frontal, zygomatic, buccal and marginal mandibular branches were identified and isolated; P2: Six FN branches were identified b/l and on the right, all were directly coapted including the frontal, zygomatic, buccal, marginal and mandibular branches	Autografts: Thoracodorsal nerve (Pt 1), greater auricular nerve (Pt 2); Nerve transfer: Masseter nerve → buccal branches (Pt 2)	FN branches (frontal, zygomatic, buccal, marginal mandibular) Masseter nerve → buccal branches (P2)	8–0 nylon interrupted sutures (Pt 1); 7–0 nylon interrupted sutures (Pt 2)	Yes (NIM-2.0 device during 2nd pt revision surgery)	N/A	N/A	N/A	Yes (evidenced by regained control of facial muscles at 6–9 months)	6 months	Pt 2: low-amplitude right facial compound muscle action potential at 11 months (indicating partial reinnervation)	Improved over time (qualitative assessment via Sunnybrook scores and photographic analysis)	Yes (graded as mild/moderate using Sunnybrook system); pt 1: synkinesis with forehead wrinkle and lip pucker; pt 2: synkinesis with smiling and lip puckering	High satisfaction mentioned (improved oral control, speech, and social reintegration) but no standardized scales (e.g., FACE-Q) were used
Assessment of Emotional Expressions after Full-Face Transplantation	Topçu et al.	2017	Case series	N/A	B/l FN trunks were coapted to those of the donor	N/A	N/A	N/A	N/A	N/A	N/A	N/A	N/A	By the end of the 2nd year/8th month	N/A	N/A	N/A	N/A
Recovery of facial expressions using functional electrical stimulation after full-face transplantation.	Topçu et al.	2018	Case series	N/A	N/A	N/A	N/A	N/A	N/A	For all 3 pts, TMG (1.25 mg/kg), PDN (initiated at 1,000 mg/day and decreased PO) were administered IO; at 7 days PO, TAC (0.2 mg/kg, serum level 15–20 ng/mL) was initiated; TMG was discontinued after the 10th day Thereafter, treatment was continued with PDN (20 mg/day), TAC and MMF (2 g/day)	N/A	N/A	N/A	N/A	Seen in 3rd pt	N/A	N/A	N/A
Image-based Analysis of Emotional Facial Expressions in Full Face Transplants	Bedeloglu et al.	2018	Case series	N/A	N/A	N/A	N/A	N/A	N/A	N/A	No	N/A	N/A	N/A	N/A	Gabor lbp analysis (photographic)	Yes, but not graded	N/A
Software-based video analysis of functional outcomes of face transplantation	Fischer et al.	2018	Case series	N/A	N/A	N/A	N/A	N/A	N/A	N/A	N/A	No	Yes, except eyebrow lift	3 months	No	Yes, except eyebrow lift, emotient software (photographic)	Yes, but not graded	N/A
The Helsinki approach to face transplantation	Lindford et al.	2019	Case series	N/A	N/A	Allograft	N/A	N/A	N/A	TMG as induction & TAC, MMF as maintenance	No, Banff consensus criteria was used as backup	Sunnybrook and Terzis scores assessed for 30 months	N/A	N/A	N/A	Yes in 1st pt but partial, slight movement in 2nd pt	N/A	N/A
Recognizing Emotional Expression as an Outcome Measure After Face Transplant.	Dorante et al.	2020	Case series	N/A	N/A	N/A	N/A	N/A	N/A	N/A	N/A	N/A	Yes, except happiness	24 months	Yes	N/A	N/A	N/A
Facial Nerve Revascularization Strategies in Facial Restoration	Khajuria et al.	2022	Case series	N/A	N/A	Allograft	N/A	N/A	N/A	No	N/A	N/A	N/A	N/A	N/A	N/A	N/A	N/A
Mini-temporalis transfer as an adjunct procedure for smile restoration	Terzis et al.	2009	Cohort study	N/A	N/A	Allograft	N/A	N/A	N/A	N/A	N/A	Yes	Yes	N/A	Yes, for 25 pts	N/A	N/A	N/A
Long-term outcomes of facial nerve function in irradiated and nonirradiated nerve grafts	Leong et al.	2013	Cohort study	Greater auricular, sural, hypoglossal, Ansa cervicalis	FN	N/A	N/A	Sep 9–0	N/A	N/A	N/A	45% of patients had an HB grade of III or IV at long-term follow-up, the best outcome (HB grade III) was observed after cross-facial grafting with sural nerve	N/A	N/A	N/A	N/A	N/A	N/A
Neuromotor Speech Recovery Across Different Behavioral Speech Modifications in Individuals Following Facial Transplantation	Eshghi et al.	2021	Cohort study	FN branches (buccal, marginal mandibular, zygomatic, frontal)	FN stump (coapted branches; exact details not specified)	N/A	Buccal, marginal mandibular, zygomatic, frontal branches	N/A	N/A	N/A	N/A	Not found (kinematic measures used: speed/range of lip/jaw movement)	N/A	N/A	Not specified (motion capture used for biomechanical analysis)	N/A	N/A	N/A
Facial Expression after Face Transplant: An International Face Transplant Cohort Comparison	Dorante et al.	2023	Cohort study	FN branches	FN branches (trunk/branch level)	Autograft, Allograft (donor nerve grafts)	At distal branch & proximal trunk level	N/A	No	N/A	N/A	HB-score: Median motor function recovery: 36.9%, Smile: 37.2%, varying by cohort	N/A	N/A	N/A	Assessed via FaceReader software—ISV comparison with controls	N/A	FDI reported outcomes for Boston cohort with mean of 69.1%

FN, facial nerve; b/l, bilateral; pt, patient; pts, patients; ATG, antithymocyte globulin (immunosuppressive induction agent); TAC, tacrolimus (calcineurin inhibitor for maintenance immunosuppression); MMF, mycophenolate mofetil (antimetabolite for immunosuppression); PDN, prednisone (oral corticosteroid); MPDN, methylprednisolone (intravenous corticosteroid); TMG, thymoglobulin (rabbit-derived polyclonal antibody preparation for induction); STR, steroids (general corticosteroid use); IL-2R Ab, interleukin-2 receptor antibody (monoclonal immunosuppressive agent); CMV, cytomegalovirus; PCP, Pneumocystis jirovecii pneumonia; EMG, electromyography; NCS, nerve conduction studies; HB, House–Brackmann grading system for facial nerve function; QOL, quality of life; MCS, mental component score of the SF-36; PO, postoperative; POD, postoperative day; IO, intraoperative; ISV, intensity score value used in facial expression analysis; FACE-Q, validated patient-reported outcome instrument for facial aesthetics and functional recovery.

### 3.3 Functional outcomes following facial nerve coaptation

Functional motor recovery following FN reconstruction was variably reported, with significant heterogeneity in outcome measures, follow-up durations, and assessment modalities.

One assessment modality was the HB Grading System. Quantitatively, the median time to first EMG-confirmed activity was 4.1 months (range: 1–6 months), and the median onset of voluntary facial motion was 5.3 months (range: 3–9 months) in cases reporting sufficient detail (*n* = 12 NRIs, 8.6%). HB grade outcomes similarly showed measurable improvement in a subset of recipients (*n* = 42): 45% of the cases in this one study achieved HB grade III–IV, and where numerical data allowed, this corresponded to an estimated 95% CI of ∼30%–61% (*n* = 14 NRIs, 11%). Spontaneous facial movement was regained with initial voluntary motion at approximately 3–6 months. postoperatively, particularly in muscles such as the levator labii, orbicularis oris, and zygomaticus major.

EMG evidence of reinnervation was typically first reported between 1 and 6 months postoperatively, with gradual improvements in amplitude and reduced latency over time. In more detailed NRIs (*n* = 44, 32%), EMG confirmed motor unit recruitment in the frontalis, orbicularis oculi, and mentalis muscles, with recovery continuing for up to 38 months. Despite partial or delayed reinnervation in *n* = 3 (2.2%) NRIs, facial symmetry at rest and during movement (smile, brow elevation) generally improved over time and was often assessed qualitatively or through photographic software (e.g., FaceReader™, Emotient™, or Gabor LBP analysis).

Moreover, synkinesis was reported in *n* = 11 (7.9%) NRI recipients, typically graded as mild to moderate and involving unintended movements during smiling or lip pursing. Patient-reported outcomes, though inconsistently collected, generally reflected high satisfaction. Positive trends were observed in domains such as oral continence, speech, social reintegration, and psychosocial wellbeing. Scales like the Facial Disability Index (FDI; i.e., a reliability and validity of a disability assessment instrument for disorders of the facial neuromuscular system), Oral Health Impact Profile (OHIP-14; i.e., a 14-item short form assessing the social impact of oral disorders on quality of life), and Short Form-36 Health Survey (SF-36; i.e., a 36-item instrument measuring general health-related quality of life across eight domains) were selectively used to quantify functional gains, although qualitative assessments predominated. Overall, these findings highlighted that while recovery is gradual and incomplete after many NRIs, substantial improvements in motor function and quality of life are attainable with appropriate nerve reconstruction strategies. Complete functional outcomes are provided in Table 2.

### 3.4 General and facial nerve–related complications

Acute graft rejection episodes were reported in *n* = 12 (8.6%) NRIs, often occurring within the first 1–2 months postoperatively and managed with steroid boluses, tacrolimus adjustments, or extracorporeal photochemotherapy. Although functional motor recovery (e.g., HB grade III–IV) was achieved, several NRIs (*n* = 69, 50%) required further procedures, including nerve transfers, interposition grafts, or cross-FN grafting due to incomplete reinnervation, asymmetric contraction, or coaptation failure. Here, secondary surgeries were common (*n* = 27, 19% NRIs), including nerve re-coaptation, hematoma evacuation, and soft tissue adjustments to optimize smile symmetry and FN branch alignment. In *n* = 12 (8.6%) NRIs, infectious complications, including CMV, HSV, and Pseudomonas-related necrosis, led to graft deterioration or systemic morbidity. Corticosteroid-related adverse effects (e.g., hyperglycemia, myalgia, osteonecrosis) were reported in *n* = 3 (2.2%) NRI recipients. Conversely, FN-specific sequelae such as neuropraxia, synkinetic overactivation, and delayed reinnervation were linked to functional deficits in *n* = 18 (13%) NRIs.

Overall, these findings emphasized the delicate interplay between immunologic control and precise microsurgical FN repair in achieving optimal FN function post-transplant (Table 3).

**Table 3:** Complications after facial nerve reconstruction.

Study title	First author	Year of publication	Follow-up time	Immunosuppression Regimen	Graft Rejection Episodes	Synkinesis Development	Need for Revision Surgery	Infection/Complications	Corticosteroid-Related Issues	Need for Additional Facial Reanimation Procedures
First human face allograft: early report	Devauchelle et al.	2006	0.33 (4 months)	ATG, TAC, MMF, PDN; donor bone marrow infusions	1 episode at day 20; grade I-II rejection; treated with steroids	N/A	N/A	Candida stomatitis (day 18); transient thrombocytosis	N/A	N/A
Technical and Anatomical Considerations of Face Harvest in Face Transplantation	Baccarani et al.	2006	N/A	N/A	N/A	N/A	N/A	N/A	N/A	N/A
Outcomes 18 months after the first human partial face transplantation	Dubernard et al.	2007	18 months	Intravenous ATG (Thymoglobulin, Genzyme) × 10 days, oral tacrolimus (trough 10–15 ng/mL, first month), mycophenolate mofetil 2 g/day, prednisone 250 mg day 1: 100 mg day 2: 60 mg/day to day 12, then tapered	Extracorporeal photochemotherapy added at month 10 to prevent rejection recurrence	N/A	N/A	Viral infection	N/A	N/A
Osteocutaneous face transplantation	Follmar et al.	2007	N/A	N/A	N/A	N/A	N/A	N/A	N/A	N/A
Facial allograft transplantation: Fiction or reality?: Sques in a fresh human cadaver model	Meßmer et al.	2008	N/A	N/A	N/A	N/A	N/A	N/A	N/A	N/A
Human facial allotransplantation: a 2-year follow-up study	Guo et al.	2008	2 years	TAC, MMF, STR, humanized IL-2 receptor MAB	3 episodes at 3, 5, 17 months; treated with TAC dose adjustment/steroid pulses	N/A	Yes (two revisions: scar revision, autologous cartilage graft for orbital floor)	Hyperglycemia (new-onset diabetes mellitus), transient thrombocytosis, dysbiosis of intestinal flora	Hyperglycemia (managed with insulin/medication)	N/A
Face Transplant Graft Procurement: A Preclinical and Clinical Study	Meningaud et al.	2008	N/A	N/A	N/A	N/A	N/A	N/A	N/A	N/A
Mini-temporalis transfer as an adjunct procedure for smile restoration	Terzis et al.	2009	Longer than 3 months	N/A	N/A	N/A	N/A	N/A	N/A	Cross-facial grafting or mini-hypoglossal-to-facial nerve
Near-total human face transplantation for a severely disfigured patient in the USA	Siemionow et al.	2009	N/A (last mentioned 5 months)	Induction: Rabbit ATG, MPDN; Maintenance: TAC, MMF. Low-dose PDN	Day 47-graft mucosa (Tx steroid bolus)	N/A	N/A	None	N/A	N/A
The Technical and Anatomical Aspects of the World's First Near-Total Human Face and Maxilla Transplant	Alam et al.	2009	N/A	N/A	N/A	N/A	N/A	N/A	N/A	N/A
Facial Transplantation: An Anatomic and Surgical Analysis of the Periorbital Functional Unit	Vasilic et al.	2010	N/A	N/A	N/A	N/A	N/A	N/A	N/A	N/A
An Update on Facial Transplantation Cases Performed between 2005 and 2010	Siemionow et al.	2010	N/A	Pt 1: Induction—ATG, MMF, prednisolone; maintenance—tacrolimus, sirolimus, MMF, prednisolone, IL-2R Ab. Pt 2: Induction—tacrolimus, MMF, MPDN, IL-2R Ab; maintenance—tacrolimus, MMF, PDN, IL-2R Ab. Pt 3: Induction—antilymphocyte serum, PDN; maintenance—tacrolimus, MMF, PDN. Pt 4: Induction—rabbit ATG, MPDN, tacrolimus; maintenance—tacrolimus, MMF, PDN	N/A	N/A	N/A	N/A	N/A	N/A
Reconstruction of a severe facial defect by allotransplantation in neurofibromatosis type 1: A case report	Sicilia-Castro et al.	2011	6 months	Induction: Basiliximab (20 mg), TAC (6 mg), MPDN. Maintenance: PDN (10 mg/d), MMF (1.5 g/d), TAC (target 8–10 ng/mL)	1, POD 28 (Banff grade III); TAC dose adjustment, MPDN pulse therapy, topical TAC	N/A	Yes (surgical revision at day 7 for hematoma evacuation)	Yes, IO: Significant blood loss (24 units packed RBCs, plasma/platelets), PO: Prerenal insufficiency (months 4–5; managed with TAC reduction).	N/A	N/A
Full Face Transplant The First Case Report	Barret et al.	2011	N/A [last mentioned 4 months (Discharge)]	Induction: TMG, PDN; PDN, TAC, MMF (switched to sirolimus)	Yes (2, MMF switched to Sirolimus)	N/A	Venous thrombosis left external jugular and left retromandibular veins-exploration and re-anastomosis	Venous thrombosis	None	N/A
Feasibility, Reproducibility, Risks and Benefits of Face Transplantation: A Prospective Study of Outcomes	Lantieria et al.	2011	0.58–3.2 years (7–38 months)	Induction: ATG, TAC, MMF; Maintenance: TAC, MMF, PDN	3 pts with 1 episode each	N/A	Yes (insufficient coaptation of right FN)	All pts (mostly bacterial; CMV; HSV; 1pt with pseudomonas aeruginosa infection led to significant necrosis, anoxic cardiac arrest after surgery 2/2 tracheotomy obstruction led to severe anoxic brain injury and eventually death)	N/A	1 pt-absence of motor recovery on the right side after 11 months led re-intervention-zygomatic muscle contraction appeared 1st, complete mouth closure by 8–12 months
Restoration of Facial Form and Function After Severe Disfigurement from Burn Injury by a Composite Facial Allograft	Pomohac et al.	2011	1,25 years	Induction: MPDN, Rabbit ATG, MMF; Maintenance: MMF & TAC	Yes, day 17 (Tx. steroids)	No	Yes (trimming of redundant cheek skin 6 months after FT)	N/A (rosacea from donor Tx. topical metronidazole)	N/A	N/A
Novel surgical technique for full face transplantation	Pomahac et al.	2012	N/A	Induction immunosuppression	No	N/A	No	No	N/A	N/A
Nerve transfers for facial transplantation: a cadaveric study for motor and sensory restoration	Audolfsson et al.	2013	N/A	N/A	N/A	N/A	N/A	N/A	N/A	N/A
Long-term outcomes of facial nerve function in irradiated and nonirradiated nerve grafts	Leong et al.	2013	2 years	N/A	N/A	N/A	No	No	No	Cross-facial grafting with sural nerve in 9th pt, greater auricular nerve in 15 pt with cable nerve graft, 16th pt had transposition nerve repair, radiotherapy in 21st pt, gold weight, botox, fat injection
Facial allotransplantation procurement using a transparotid approach: A new anatomical model	Horta et al.	2014	N/A	N/A	N/A	N/A	N/A	N/A	N/A	N/A
A functional periorbital subunit allograft: Vascular, anatomic, and technical considerations for future subunit facial transplantation	Mathes et al.	2014	N/A	N/A	N/A	N/A	N/A	N/A	N/A	N/A
Eyelid Transplantation: Lessons from a Total Face Transplant and the Importance of Blink	Sosin et al.	2015	1,1 years	Not specified (corticosteroids mentioned in discussion)	N/A	N/A	Yes (Le Fort III advancement, brow elevation at 6 months; b/l lower eyelid blepharoplasty at 9 months)	Temporary neuropraxia, corneal exposure post-revision; tx: artificial tears	N/A	N/A
Long-Term Multifunctional Outcome and Risks of Face Vascularized Composite Allotransplantation	Roche et al.	2015	3 years	Maintenance therapy of corticoids, TAC, MMF in minimal doses	Rejection successfully treated	N/A	N/A	Myalgia, aspergilloma	N/A	N/A
Referred facial sensation on the hand after full face transplantation	Uysal et al.	2016	2 years	Induction: ATG, PDN; Maintenance: TAC, MMF, PDN	N/A	N/A	N/A	III-defined pain sensation on the face, incomplete recovery of emotional facial expressions	N/A	N/A
Surgical Optimization of Motor Recovery in Face Transplantation	Aycart et al.	2016	3,5 years	N/A	N/A	Yes (graded as mild/moderate using Sunnybrook system)	Pt 2: Yes (nerve transfer at 11 months post-transplant due to impaired motor recovery)	N/A	N/A	Yes (Pt 2 required nerve transfer and interposition graft)
						Pt 1: Synkinesis with forehead wrinkle and lip pucker				
						Pt 2: Synkinesis with smiling and lip puckering				
The First Immediate Face Transplant in the World	Maciejewski et al.	2016	N/A (2 yr documented)	Induction: ATG, TAC, MMF, MPDN; Maintenance: TAC, MMF, MPDN	Yes [POD 34 (Grade 2 histopathology), Tx steroids]	N/A	N/A	N/A	N/A	N/A
Facial nerve regeneration after facial allotransplantation: A longitudinal clinical and electromyographic follow-up of lip movements during speech	De Letter et al.	2017	3 years	N/A	Yes (1 episode at 4 months, treated with immunosuppressants)	N/A	N/A	Yes (Aspergillus infection at 12 months PO, treated)	N/A	N/A
The Effects of Lip-Closure Exercise on Lip Strength and Function Following Full Facial Transplantation: A Case Report	Bridget et al.	2017	1 year	N/A	N/A	N/A	N/A	N/A	N/A	Yes, an 8-week targeted lip-strengthening biofeedback program was trialed with positive results
Assessment of Emotional Expressions after Full-Face Transplantation	Topçu et al.	2017	1–2 years	N/A	N/A	N/A	N/A	N/A	N/A	N/A
Recovery of facial expressions using functional electrical stimulation after full-face transplantation	Topçu et al	2018	N/A	For all 3 pts, TMG (1.25 mg/kg) and prednisolone (initiated at 1,000 mg/day and decreased PO) were administered during the surgery. At 7 days PO, TAC (0.2 mg/kg, serum level 15–20 ng/mL) was initiated. TMG was discontinued after the 10th day. Thereafter, treatment was continued with PDN (20 mg/day), TAC, and MMF (2 g/day)	N/A	N/A	N/A	N/A	N/A	N/A
Image-based Analysis of Emotional Facial Expressions in Full Face Transplants	Bedeloglu M	2018	3–4 years	N/A	N/A	Yes, but not graded	N/A	N/A	N/A	Rehabilitation still going on
Software-based video analysis of functional outcomes of face transplantation	Fischer et al.	2018	1 year	N/A	N/A	Yes, but not graded	N/A	N/A	N/A	N/A
The Helsinki approach to face transplantation	Lindford et al.	2019	30,5 months	TMG as induction & TAC, MMF as maintenance	No, Banff consensus criteria used	N/A	Yes	Nasopalatine fistula, EBV, CMV infection (in 1st pt) sialocele, oronasal fistula, palatine necrosis (in 2nd pt)	Diabetes	No
Recognizing Emotional Expression as an Outcome Measure After Face Transplant	Dorante et al.	2020	2 years	N/A	N/A	N/A	N/A	N/A	N/A	Yes
Full facial retransplantation in a female patient—Technical, immunologic, and clinical considerations	Kauke et al.	2021	0,5 years post re-transplantation (7 years 3 months total from first transplant)	Induction: ATG, MMF, MPDN; Maintenance: PDN (10 mg QD), TAC (3 mg BID, goal 8–10 ng/mL), MMF (1,000 mg BID); Prophylaxis: Valganciclovir (CMV), trimethoprim-sulfamethoxazole (PCP)	1st transplant: Chronic antibody-mediated rejection (AMR) and recurrent T cell-mediated rejection (TCMR), leading to irreversible graft loss at 88 months. Retransplant: Grade III TCMR at 3 and 4 months post-retransplant, treated with STR and alemtuzumab	N/A	No	CMV viremia post-retransplant (treated with valganciclovir) significant intraoperative bleeding (2.5 L blood loss)	N/A	N/A
Neuromotor Speech Recovery Across Different Behavioral Speech Modifications in Individuals Following Facial Transplantation	Eshghi et al.	2021	Early group: 2 months; Late group: 42 months	N/A	N/A	N/A	N/A	N/A	N/A	N/A
Face Transplant: Current Update and First Canadian Experience	Govshievich et al.	2021	18 months	N/A	N/A	N/A	N/A	Mucormycosis of left thigh	N/A	N/A
Facial Nerve Revascularization Strategies in Facial Restoration	Khajuria et al.	2022	6 years	No requirement	N/A	N/A	N/A	Flap necrosis, hematoma	N/A	No
Re-cognizing the new self: The neurocognitive plasticity of self-processing following facial transplantation	Azevedo et al.	2022	2 years	N/A	N/A	N/A	Yes [repair of floor-of-mouth & palatal wound dehiscence on POD 11, internal fixation of left mandibular nonunion, b/l canthoplasty & complex tissue rearrangement lower eyelids & cheeks (POD 108), left medial canthoplasty & complex tissue rearrangement of left lower eyelid (POD 248))	N/A	N/A	N/A
Facial Expression after Face Transplant: An International Face Transplant Cohort Comparison	Dorante et al.	2023	3.6 years ± 2.2 months	N/A	N/A	N/A	Yes, nerve transfer revision	N/A	N/A	Yes, e.g., masseter-to-facial nerve transfer
Anatomical study of trigeminal-facial nerve communications: Application to facial transplant surgery	Iwai et al.	2025	N/A	N/A	N/A	N/A	N/A	N/A	N/A	N/A

N/A, not available/not applicable; m, male; f, female; FN, facial nerve; ATG, antithymocyte globulin; TAC, tacrolimus; MMF, mycophenolate mofetil; PDN, prednisone; STR, steroid therapy; MAB, monoclonal antibody; MPDN, methylprednisolone; Tx, treatment; Pt/Pts, patient/patients; Ab, antibody; TMG, Thymoglobulin; POD, postoperative day; IO, intraoperative; PO, postoperative; CMV, cytomegalovirus; HSV, herpes simplex virus; FT, facial transplantation; Sx, surgery; b/l, bilateral; Le Fort III, anatomical classification of midface fracture used for orientation in facial surgery; PCP, Pneumocystis pneumonia; BID, twice daily.

### 3.5 Perspectives and preclinical advances

Preclinical evidence was scarce and limited to rodent and porcine models. Overall, study results supported the clinical evidence that meaningful FN regeneration can be achieved when both motor and sensory nerves are coapted. In a rat model, vascularized mystacial pad flaps transplanted across a full MHC mismatch demonstrated successful reinnervation when motor (buccal, marginal mandibular, zygomatic) and sensory (infraorbital) nerves were repaired. These flaps exhibited restored whisker-defense reflexes, ENG amplitudes around 2 mV, and myelinated fibers on histology six weeks postoperatively, while non-repaired controls showed no electrical activity. In a hemiface transplant model of rats, only grafts with both FN branch and infraorbital coaptation demonstrated motor potentials and cortical activity in the barrel cortex, whereas denervated flaps showed none. At last, one study using a heterotopic midface transplant (nose, premaxilla, and lip) of rats with nerve coaptation showed long-term survival (>100 days) in both isografts and immunosuppressed allografts. These exhibited somatosensory- and motor-evoked potential latencies reaching 67% and 70% of native values, respectively, alongside viable bone on CT (Table 4).

**Table 4:** Preclinical evidence on facial nerve reconstruction in facial VCA.

DOI	Title	Author	Year of publication	Study type	Animal model	Intervention	Objective of intervention	Comparison groups	Outcome
DOI: 10.1111/j.1432-2277.2009.01032.x	A new composite midface allotransplantation model with sensory and motor reinnervation	Zor et al.	2009	*In-vivo*	Inbred rats (8- to 10-week-old); recipient: Lewis (RT1^l), donor: Lewis (RT1^l) and Lewis-Brown Norway (LBN, RT1^l + n)	Composite midface graft (including nose, lower lip, masseter, and premaxilla with hard palate and teeth) was harvested on the donor's carotid artery and jugular vein, incorporating both infraorbital (sensory) and FN (motor) branches; heterotopic transplantation to the recipient's inguinal region with vascular anastomoses to femoral vessels and nerve coaptations (infraorbital to saphenous, FN to femoral); cyclosporine A monotherapy	Extension of the rat face transplant model via a composite midface allograft including infraorbital and facial nerves plus bone and soft tissue, enabling long-term functional recovery assessment through CT, SSEP, and MEP	Group I: Anatomic study (model development/dissection, *n* = 3); Group II: Isograft transplants (between genetically identical Lewis rats, *n* = 5); Group III: Allograft transplants (from Lewis-Brown Norway donors to Lewis recipients under CsA monotherapy, *n* = 5)	All composite midface grafts survived >100 days with confirmed vascularization (microangiography) and bone viability (CT). Motor function returned by day 20 and reached ∼70% of normal FN values; sensory recovery achieved ∼67% of normal ION latencies. Imaging and electrophysiology confirmed successful reinnervation and functional restoration
DOI: 10.1097/SAP.0b013e31819031ef	Sensorimotor recovery after partial facial (mystacial pad) transplantation in rats	Landin et al.	2009	*In-vivo*	Inbred rats; recipient: Wistar-Lewis (RT1 L), donor: Lewis-Brown-Norway (RT1ln)	Partial facial transplantation using a mystacial pad flap based on facial vessels, with microvascular anastomoses; two study groups: i) with nerve repair (including bucolabial, marginal mandibular, zygomatico-orbital FN branches and the infraorbital nerve) and ii) without nerve repair	Investigation of nerve repair's impact on sensorimotor recovery in mystacial pad allotransplants, using clinical, neurophysiological, and histologic outcome measures	Multiple experimental groups were created, with the main comparison between non-neurotized alloflaps (VIa) and neurotized alloflaps with nerve repair (VIb); additional isograft and flap groups assessed graft viability and take	Average operative time was ∼3.5 h with an 87.5% survival rate at 8 weeks. Flaps with nerve repair (VIb) showed significant sensorimotor recovery on ENG, EMG, and histology, while non-repaired flaps (VIa) showed signs of denervation
DOI: 10.1097/prs.0b013e318191bca2	A model for functional recovery and cortical reintegration after hemifacial composite tissue allotransplantation	Washington et al.	2009	*In-vivo*	Inbred rats (8- to 10-week-old); recipient: Lewis (RT1 L), donor: Brown-Norway (RT1n)	Hemifacial transplant including mystacial pad with microsurgical vascular anastomoses and motor (buccal, marginal mandibular) and sensory (infraorbital) nerve coaptations; groups with and without nerve repair; allografts received cyclosporine A	Development of a functional rat hemifacial transplant model that allows studying motor and sensory recovery—including cortical reintegration	Group 1: Syngeneic transplants with motor and sensory nerve appositions; Group 2: Syngeneic transplants without nerve appositions; Group 3: Allogeneic transplants with nerve appositions (with cyclosporine A immunosuppression)	Groups with nerve appositions (1 and 3) showed significant motor (whisking, conduction) and sensory (cortical response) recovery, while non-neurotized grafts (group 2) lacked electrical or cortical activity
DOI: 10.1097/SCS.0000000000002449	Surgical Technique of Hemi-Face Transplant: A New Model of Training	Cunico et al.	2016	*In-vivo*	Seven swines (Sus scrofa domesticus, Landrace line, approximately 60 days old and weighing between 10 and 20 kilograms)	Excision and immediate reimplantation of the left hemiface at the same site using microsurgical vascular and nerve anastomoses under magnification	Development of a reproducible swine hemifacial transplant model for surgical training, focusing on microsurgical reconstruction of vessels and nerves	Comparison of immediate post-euthanasia procedures vs. delayed procedures after cooling to evaluate differences in hemostasis and tissue handling	The procedure averaged 4.5 h with consistent reproducibility; anatomical and technical challenges—such as obesity and vascular variations (e.g., caudal auricular artery)—were noted, confirming the model's suitability for microsurgical training

DOI, Digital Object Identifier; FN, Facial Nerve; CT, Computed Tomography; SSEP, Somatosensory-Evoked Potential; MEP, Motor-Evoked Potential; ENG, Electroneurography; EMG, Electromyography; ION, Infraorbital Nerve; CsA, Cyclosporine A.

## 4 Discussion

FN reconstruction is a critical determinant of functional success in FVCA. While surgical advancements have rendered full or partial face transplantation technically feasible, the restoration of dynamic facial expression remains one of the most complex and unpredictable aspects of the procedure (32). Emerging patterns from the available evidence suggest a preliminary, clinically relevant framework in which allograft extent, reconstructive strategy, neuromuscular recovery phase, and immunologic stability function as interdependent domains shaping postoperative outcomes. The intricate nature of FN injury and repair in FVCA necessitates individualized coaptation strategies, including direct repair, nerve grafting, and targeted nerve transfers, to address anatomical and physiological challenges (33). Unlike conventional facial nerve surgery, FVCA involves donor–recipient anatomical mismatches, variable nerve diameters, and the need to coordinate reinnervation across multiple composite tissue units. Additionally, immunologic factors unique to allotransplantation, such as rejection episodes and the effects of long-term immunosuppression, can directly influence nerve regeneration and graft viability. The requirement to achieve both motor and sensory reinnervation across a transplanted facial framework further compounds the complexity of achieving predictable functional outcomes. Therefore, this discussion aims to compare FN reconstruction in FVCA to established approaches in conventional FN and peripheral nerve repair and provide a critical analysis of the spectrum of techniques employed in both clinical and preclinical studies. Thereby, this review seeks to highlight current outcomes and explore emerging strategies to enhance reinnervation and optimize long-term functional recovery of the FN following FVCA.

**Figure 2:**
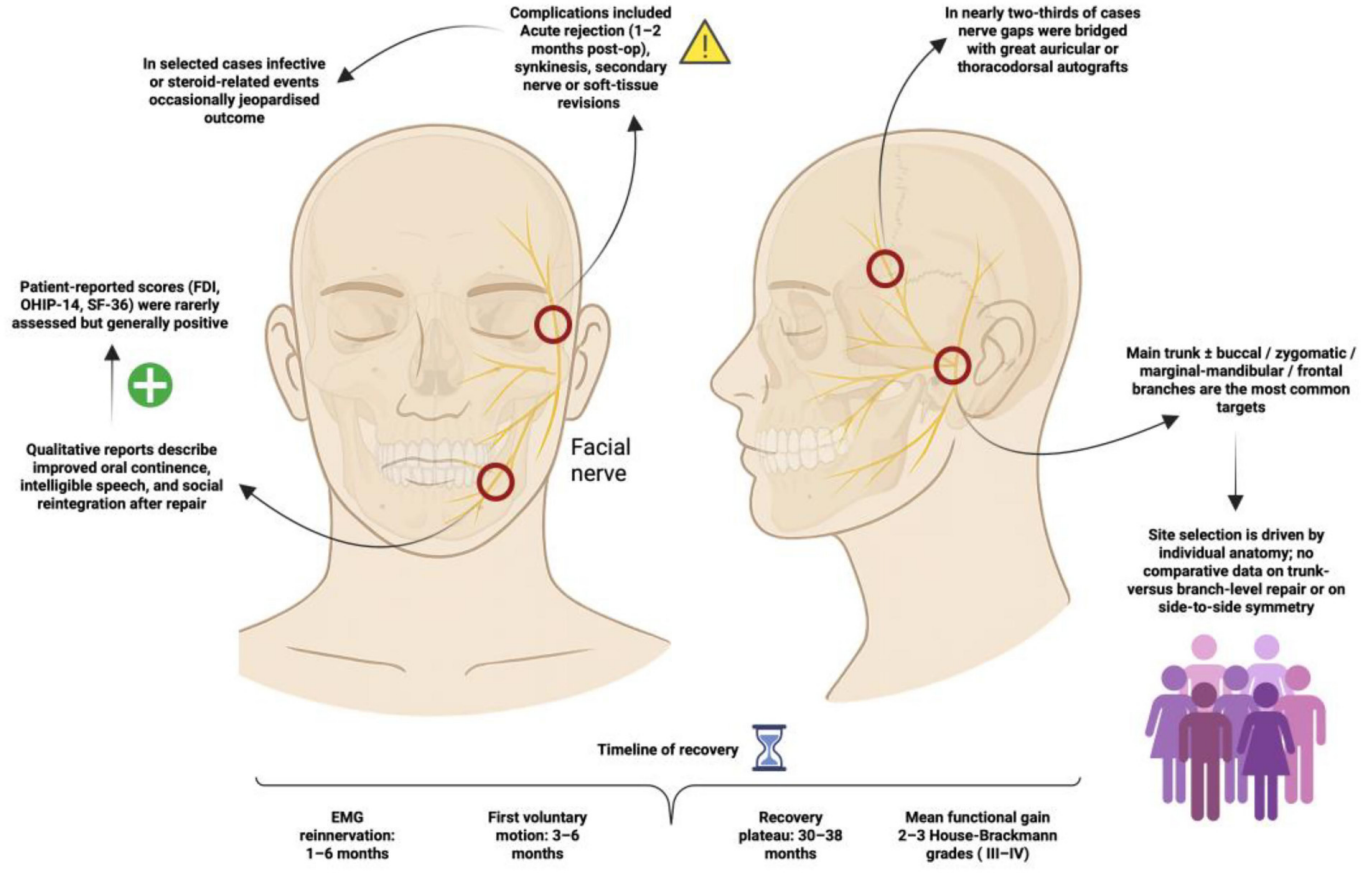
Facial-nerve repair in facial VCA—techniques, recovery arc, and clinical impact: direct branch-to-branch coaptation is attempted when feasible, but autografts bridge gaps in roughly two-thirds of facial VCAs; most repairs target the main trunk or its buccal/zygomatic/marginal-mandibular/frontal branches. EMG activity typically returns within 1–6 months, voluntary motion follows by 3–6 months, and function plateaus at 30–38 months—typically improving two to three House-Brackmann grades (ending at III–IV). Benefits include better oral continence, intelligible speech and social reintegration, yet complications—acute rejection, synkinesis and revision surgery—remain common and might blunt overall gains.

In our study, we found direct coaptation of FN branches to be the most common repair strategy, often resulting in partial functional recovery within as little as 3–6 months. EMG evidence supported gradual reinnervation, though outcomes varied, and synkinesis or revision procedures were occasionally required. Surgical complications further influenced long-term FN function, highlighting the need for refined FN reconstruction techniques and outcome assessment methods in FVCA (Figure 2).

When comparing this to literature, FN reconstruction in FVCA presents unique technical and biological challenges that distinguish it from conventional FN and peripheral nerve repair (2–4, 34).

### 4.1 Technical factors influencing recovery

FN reconstruction in FVCA presents distinct technical challenges compared with conventional FN or peripheral nerve repair. In standard FN surgery, such as after trauma or oncologic resection, tension-free, end-to-end coaptation remains the gold standard and typically yields meaningful recovery within 3–6 months (35, 36). When direct coaptation is not feasible, interpositional autografts (sural or great auricular nerve) or motor nerve transfers (hypoglossal–FN, masseteric–FN) are well-established options, with many patients achieving HB III–IV function (37–39). In FVCA, however, the reconstructive environment is inherently more complex. Surgeons must contend with donor–recipient anatomical mismatch, variable branch orientation, and the need to integrate nerves within a composite tissue allograft (18, 40, 41). Although direct coaptation remains preferred when feasible, the risk of misalignment or distal branch mismatch is greater than in isolated FN reconstruction, even more so when interpositional grafts are required (42–44). Donor nerves also traverse composite soft tissue and skeletal components, making successful recovery dependent not only on microsurgical precision but also on the viability and integration of the transplanted neuromuscular units (45, 46). Recovery timelines differ accordingly. While conventional FN repairs often show substantial motor recovery within 6–12 months, FVCA recovery is more variable. Initial motion may occur around 3–6 months, but EMG evidence suggests that reinnervation may continue for 24–36 months or longer (47, 48). Prolonged recovery likely reflects greater regenerative distances, pre-existing scarring, and delayed reconstruction as well as technical and biologic constraints unique to FVCA (49, 50).

### 4.2 Immunological factors influencing recovery

Complications such as synkinesis, asymmetric movement, and incomplete motor recovery are common to both standard FN repair and VCA. However, immunologic dynamics represent one of the most consequential differences between FVCA and conventional FN repair (51). FVCA recipients frequently experience acute or subclinical rejection episodes in the early postoperative period, often treated with high-dose steroids or adjustments to tacrolimus therapy (22, 52, 53). Complications such as synkinesis, asymmetric movement, and incomplete activation occur in both settings, but in FVCA these issues may be compounded by rejection-related injury or ischemic episodes. As a result, revision procedures, including nerve re-coaptation, static suspension, or supplementary reanimation techniques, are required more frequently in FVCA than conventional nerve repairs (54). Thus, unlike isolated FN repair, functional recovery in FVCA depends on achieving and maintaining not only microsurgical success but also long-term immunologic stability of the transplanted neuromuscular tissue (55).

### 4.3 Rehabilitative and outcome-assessment factors

Despite the surgical and immunologic complexity, outcome measurements in FVCA remained inconsistent. Unlike standard FN repair, where validated scales such as the HB grading system, Sunnybrook Facial Grading System (i.e., a recognized tool for assessing facial palsy with a total composite score between 0 and 100), and FDI are routinely used, VCA literature often relies on qualitative assessments or unvalidated photographic analysis. Broader implementation of validated scales, such as the FDI and facial tracking software such as FaceReader™, Emotrics™ or Emotient™, would allow for more objective and reproducible assessment of motor recovery and patient satisfaction (56–58). Additionally, digital tools like the eFACE scale have shown promise as intuitive, clinician-friendly instruments for standardized facial function evaluation across platforms (59). In conclusion, literature highlighted that while FN reconstruction in FVCA borrows from established principles in peripheral and FN surgery, it requires significant adaptation to the immunologic and anatomical complexities of composite tissue transplantation. Furthermore, optimizing surgical outcomes depended on precise microsurgical technique, consistent intraoperative neuromonitoring, and long-term rehabilitative strategies in both FVCA and conventional FN reconstruction.

### 4.4 Emerging patterns and toward a clinically actionable framework

Despite the heterogeneity of available evidence, several higher-order themes emerge that may inform a preliminary framework for understanding facial nerve reconstruction in FVCA. First, across studies, nerve coaptation strategy, whether direct, graft-assisted, or dual-level, appears consistently aligned with the extent of allograft complexity, suggesting a pattern in which more extensive transplants necessitate more elaborate reconstructive algorithms. Second, functional recovery trajectories demonstrate a relatively stable temporal pattern: early EMG activity typically emerges around 1–6 months, voluntary motion around 3–9 months, and continued maturation up to 3 years, indicating a predictable multi-phased recovery course that may aid in clinical counseling and postoperative planning. Third, cases with integrated motor and sensory coaptation (both in humans and preclinical models) generally exhibit more robust reinnervation, hinting at a potential “sensorimotor synergy” that warrants further exploration as a guiding reconstructive principle. Fourth, complication profiles consistently underscore the interplay between immunologic stability and the durability of nerve repair, suggesting that FN-related outcomes may benefit from risk-stratified immunosuppression and early detection strategies for rejection. Together, these themes suggest an emerging conceptual framework in which (1) allograft extent, (2) reconstructive strategy, (3) neuromuscular recovery phase, and (4) immunologic stability function as interdependent domains shaping outcomes. Although preliminary, this pattern-based synthesis may serve as the basis for future standardized reporting, comparative studies, and the development of actionable treatment algorithms in facial nerve reconstruction following FVCA. Linking specific reconstructive approaches to detailed functional outcomes in future studies might further strengthen this framework and help lay the groundwork for targeted investigations evaluating the effectiveness of distinct surgical strategies.

### 4.5 Summary and outlook

Moving forward, several strategies could potentially address the current challenges in FN reconstruction after FVCA. Principles from standard FN and peripheral nerve repair, such as direct coaptation, nerve grafting, and motor nerve transfers, should further be successfully adapted to the VCA setting if they are carefully tailored to the specific anatomical and immunologic environment of the transplant. In this context, innovative approaches like “supercharging”, as recently demonstrated in the Epta-innervation technique using up to seven donor nerves, may offer additional benefits in enhancing reinnervation and improving symmetry in mimetic function (60). Preoperative planning with detailed donor–recipient nerve matching and intraoperative nerve stimulation may enhance surgical precision and improve initial outcomes. Recovery trajectories in FVCA might be improved through early postoperative rehabilitation, including facial retraining, functional electrical stimulation, and targeted biofeedback. These strategies, which are well established in conventional FN rehabilitation, could support more coordinated and symmetric reinnervation (61).

At the same time, confounding factors unique to VCA, particularly the impact of systemic immunosuppression on nerve healing (e.g., tacrolimus), must be considered (62). Immunosuppressive regimens, while necessary to prevent graft rejection, might impair axonal regeneration and synaptic plasticity. Future modifications, such as localized immunosuppression or novel immunomodulatory protocols, could help mitigate these effects, although more evidence is needed. Moreover, translational research, including preclinical animal models, cadaveric nerve mapping studies, and advanced imaging analyses, might provide valuable insights into optimizing nerve coaptation strategies and improving functional outcomes. Promising clinical data also support the use of connector-assisted allograft techniques, such as Avance® nerve allografts combined with AxoGuard® sleeves, which have demonstrated high rates of functional sensory recovery, particularly in immediate reconstruction of the inferior alveolar nerve following mandibular resection (63). Establishing multicenter registries and applying standardized outcome measures, such as the FDI scale, and EMG tracking, could enable more reliable comparisons across centers and support more individualized, evidence-based treatment planning. In parallel, future strategies should prioritize the identification of predictive factors for favorable or poor outcomes, such as patient-specific variables, surgical timing, or nerve gap characteristics, which could guide clinical decision-making and help stratify patients for tailored interventions. Ultimately, careful adaptation of established surgical principles, combined with advances in immunologic management and preclinical research, could lead to more predictable nerve regeneration and better long-term facial function for patients undergoing FVCA.

## 5 Limitations

This systematic review has several limitations that must be acknowledged. First, the inherent heterogeneity of included studies limited the ability to perform a quantitative meta-analysis. Variability in study design, nerve reconstruction techniques, outcome assessment tools, and reporting time points posed challenges for direct comparisons and synthesis. Rehabilitation protocols also differed substantially across studies, further contributing to variability in reported outcomes. Additionally, a significant proportion of included studies were case reports or small case series, which introduces selection bias and limits the generalizability of findings. Second, the methodological quality of included clinical studies was overall moderate, with many lacking prospective data collection, standardized outcome measures, or comprehensive follow-up. The use of diverse and sometimes non-validated tools to assess functional recovery, such as subjective photographic analysis or qualitative descriptions, may have introduced measurement bias and prevented robust comparisons. Moreover, long-term electromyographic follow-up was rarely standardized or consistently reported, limiting the ability to compare reinnervation trajectories across interventions. In addition, many studies did not report motor and sensory outcomes separately or with sufficient detail, preventing a systematic distinction between these domains despite their relevance to comprehensive facial nerve reconstruction. Similarly, insufficient reporting on reconstructive strategies in relation to functional outcomes limited our ability to meaningfully correlate technique selection with recovery patterns, representing an important area for improvement in future studies. Third, donor and recipient characteristics were often incompletely reported, particularly regarding nerve diameter match, injury chronicity, and delay from injury to transplantation. These variables could substantially influence reinnervation success but were not systematically addressed. Similarly, the impact of immunosuppressive regimens on nerve regeneration could not be assessed due to inconsistent reporting of dose, duration, and complications. Fourth, the review may have been subject to publication bias, as negative or poor-outcome cases are less frequently published, especially in high-impact journals. This could lead to an overestimation of the effectiveness of certain surgical strategies. Finally, while efforts were made to include all relevant literature, it is possible that some pertinent studies were missed due to limitations in database indexing or language restrictions. Although the search strategy was broad and supplemented by manual reference checks, only English-language, peer-reviewed publications were included. Future reviews may benefit from international registry data, standardized reporting templates, prospective multicenter studies, and harmonized rehabilitation and EMG follow-up protocols to improve the quality, reproducibility, and comparability of findings.

## 6 Conclusion

FN reconstruction is a key determinant of functional success in FVCA. This review highlights the predominance of direct coaptation and the gradual integration of advanced techniques such as motor nerve transfers and dual level coaptation. While outcomes are encouraging, they remain variable and are shaped by surgical precision, immunologic factors, and rehabilitation. FN reinnervation is often achievable but tends to be partial and delayed. Greater use of standardized assessment tools—such as the HB Grading System, FDI, and EMG—could improve comparability across studies. Conventional nerve repair strategies may be adapted to the FVCA setting with thoughtful anatomical and immunologic tailoring. Progress will depend on translational research to understand nerve healing under immunosuppression, optimize coaptation protocols, and validate rehabilitation strategies. Multicenter data, harmonized outcome reporting, and preclinical models will be essential for advancing FN repair and improving long-term function and quality of life after FVCA.

## Data Availability

The original contributions presented in the study are included in the article/Supplementary Material, further inquiries can be directed to the corresponding author.
