# Supplementary material for: Rewiring faces: advances and outcomes in facial nerve reconstruction after facial vascularized composite allotransplantation
Source: Front Surg. 2026 Jan 30;13:1738957. doi: 10.3389/fsurg.2026.1738957 (PMC12901399; doi:10.3389/fsurg.2026.1738957)
Supplement: Supplementary file 1 [file Table1.docx]

**Supplemental digital content**

**Supplemental digital content 1.** Full search strategy for each database included

| **Database** | **Search string** | **Results** |
| --- | --- | --- |
| PubMed/MEDLINE | ("facial nerve"[MeSH Terms] OR "facial nerve"[All Fields] OR "facial nerve injury"[All Fields] OR "facial nerve regeneration"[All Fields] OR "facial nerve dysfunction"[All Fields] OR "cranial nerve VII"[All Fields])  AND  ("face transplantation"[MeSH Terms] OR "face transplantation"[All Fields] OR "facial allotransplantation"[All Fields] OR "face allograft"[All Fields] OR "facial transplant"[All Fields] OR "facial reconstructive surgery"[All Fields] OR "facial grafting"[All Fields]) | 37 |
| Web of Science | TS=("facial nerve" OR "facial nerve injury" OR "facial nerve regeneration" OR "facial nerve dysfunction" OR "cranial nerve VII")  AND  TS=("face transplantation" OR "facial allotransplantation" OR "face allograft" OR "facial transplant" OR "facial reconstructive surgery" OR "facial grafting") | 33 |
| EMBASE | ('facial nerve'/exp OR 'facial nerve' OR 'facial nerve injury' OR 'facial nerve regeneration' OR 'facial nerve dysfunction' OR 'cranial nerve VII')  AND  ('face transplantation'/exp OR 'face transplantation' OR 'facial allotransplantation' OR 'face allograft' OR 'facial transplant' OR 'facial reconstructive surgery' OR 'facial grafting') | 80 |
| Google Scholar | ("facial nerve" OR "facial nerve injury" OR "facial nerve regeneration" OR "facial nerve dysfunction" OR "cranial nerve VII")  AND  ("face transplantation" OR "facial allotransplantation" OR "face allograft" OR "facial transplant" OR "facial reconstructive surgery" OR "facial grafting") | 250 |
| Cochrane | ("facial nerve" OR "facial nerve injury" OR "facial nerve regeneration" OR "facial nerve dysfunction" OR "cranial nerve VII")  AND  ("face transplantation" OR "facial allotransplantation" OR "face allograft" OR "facial transplant" OR "facial reconstructive surgery" OR "facial grafting") | 2 |

**Supplemental digital content 2.** Quality assessment of studies included via the Level of Evidence scoring system

| **DOI** | **Study title** | **First author** | **Year of publication** | **Study type** | **Level of Evidence** |
| --- | --- | --- | --- | --- | --- |
| DOI: 10.1016/S0140-6736(06)68935-6 | First human face allograft: early report | Devauchelle et al. | 2006 | Case report | 4 |
| DOI: 10.1097/01.sap.0000227486.28556.3e | Technical and Anatomical Considerations of Face Harvest in Face Transplantation | Baccaraniet al. | 2006 | Cadaveric study | 5 |
| DOI: 10.1056/NEJMoa072828. | Outcomes 18 months after the first human partial face transplantation. | Dubernard et al. | 2007 | Case report | 4 |
| DOI:10.1016/j.bjps.2007.12.014 | Osteocutaneous face transplantation | Follmar et al. | 2007 | Cadaveric study | 5 |
| DOI:10.1007/s00104N/A007-1446-x | Facial allograft transplantation: Fiction or reality? : Sques in a fresh human cadaver model | Meßmer et al. | 2008 | Cadaveric study | 5 |
| DOI:10.1016/S0140-6736(08)61276-3 | Human facial allotransplantation: a 2-year follow-up study | Guo et al. | 2008 | Case report | 4 |
| DOI: 10.1097/PRS.0b013e3181882146 | Face Transplant Graft Procurement: A Preclinical and Clinical Study | Meningaud et al. | 2008 | Cadaveric study and case report | 5 |
| DOI: 10.1097/PRS.0b013e3181954e8c | Mini-temporalis transfer as an adjunct procedure for smile restoration | Terzis et al. | 2009 | Cohort study | 3 |
| DOI: 10.1016/S0140-6736(09)61155-7 | Near-total human face transplantation for a severely disfigured patient in the USA | Siemionow et al. | 2009 | Case report | 4 |
| DOI: 10.1001/archfacial.2009.80 | The Technical and Anatomical Aspects of the World's First Near-Total Human Face and Maxilla Transplant | Alam et al. | 2009 | Case report | 4 |
| DOI:10.1097/prs.0b013e3181c2a5cc | Facial Transplantation: An Anatomic and Surgical Analysis of the Periorbital Functional Unit | Vasilic et al. | 2010 | Cadaveric study | 5 |
| DOI: 10.1097/PRS.0b013e318230c77b | An Update on Facial Transplantation Cases Performed between 2005 and 2010 | Siemionow et al. | 2010 | Case series | 4 |
| DOI:10.1016/j.transproceed.2011.06.030 | Reconstruction of a severe facial defect by allotransplantation in neurofibromatosis type 1: A case report | Sicilia-Castro et al. | 2011 | Case report | 4 |
| DOI: 10.1097/SLA.0b013e318226a607 | Full Face Transplant The First Case Report | Barret et al. | 2011 | Case report | 4 |
| DOI: 10.1111/j.1600-6143.2010.03406.x | Feasibility, Reproducibility, Risks and Benefits of Face Transplantation: A Prospective Study of Outcomes | Lantieri et al. | 2011 | Case series | 4 |
| DOI:10.1111/j.1600-6143.2010.03368.x | Restoration of Facial Form and Function After Severe Disfigurement from Burn Injury by a Composite Facial Allograft | Pomohacet al. | 2011 | Case report | 4 |
| DOI: 10.1097/PRS.0b013e31825dc25c | Novel surgical technique for full face transplantation | Pomahac et al. | 2012 | Case series | 4 |
| DOI:10.1097/prs.0b013e31828bd394 | Nerve transfers for facial transplantation: a cadaveric study for motor and sensory restoration. | Audolfsson et al. | 2013 | Cadaveric study | 5 |
| DOI: 10.1177/000348941312201106 | Long-term outcomes of facial nerve function in irradiated and nonirradiated nerve grafts | Leong et al. | 2013 | Cohort study | 3 |
| DOI:10.1002/micr.22216 | Facial allotransplantation procurement using a transparotid approach: A new anatomical model | Horta et al. | 2014 | Cadaveric study | 5 |
| DOI:10.1016/j.bjps.2014.05.046 | A functional periorbital subunit allograft: Vascular, anatomic, and technical considerations for future subunit facial transplantation | Mathes et al. | 2014 | Cadaveric study | 5 |
| DOI:10.1097/prs.0000000000000798 | Eyelid Transplantation: Lessons from a Total Face Transplant and the Importance of Blink | Sosin et al. | 2015 | Case report | 4 |
| DOI: 10.1097/SCS.0000000000002110. | Long-Term Multifunctional Outcome and Risks of Face Vascularized Composite Allotransplantation | Roche et al. | 2015 | Case report | 4 |
| DOI:10.1212/WNL.0000000000002409 | Referred facial sensation on the hand after full face transplantation | Uysal et al. | 2016 | Case report | 4 |
| DOI:10.1097/SCS.0000000000002305 | Surgical Optimization of Motor Recovery in Face Transplantation | Aycart et al. | 2016 | Case series | 4 |
| DOI: 10.1097/SLA.0000000000001597 | The First Immediate Face Transplant in the World | Maciejewski et al. | 2016 | Case report | 4 |
| DOI:10.1016/j.bjps.2017.02.025 | Facial nerve regeneration after facial allotransplantation: A longitudinal clinical and electromyographic follow-up of lip movements during speech | De Letter et al. | 2017 | Case report | 4 |
| DOI:10.1044/2017_AJSLP-16-0101 | The Effects of Lip-Closure Exercise on Lip Strength and Function Following Full Facial Transplantation: A Case Report | Bridget al. | 2017 | Case report | 4 |
| DOI:10.1155/2017/8789724 | Assessment of Emotional Expressions after Full-Face Transplantation | Topçu et al. | 2017 | Case series | 4 |
| DOI: 10.1186/s12984-018-0356-0. | Recovery of facial expressions using functional electrical stimulation after full-face transplantation. | Topçu et al. | 2018 | Case series | 4 |
| DOI: 10.1007/s10916-018-0895-8. | Image-based Analysis of Emotional Facial Expressions in Full Face Transplants | Bedeloglu et al. | 2018 | Case series | 4 |
| DOI:10.1002/micr.30360 | Software-based video analysis of functional outcomes of face transplantation | Fischer et al. | 2018 | Case series | 4 |
| DOI: 10.1016/j.bjps.2018.08.030 | The Helsinki approach to face transplantation | Lindford et al. | 2019 | Case series | 4 |
| DOI: 10.1001/jamanetworkopen.2019.19247. | Recognizing Emotional Expression as an Outcome Measure After Face Transplant. | Dorante et al. | 2020 | Case series | 4 |
| DOI:10.1111/ajt.16696 | Full facial retransplantation in a female patient—Technical, immunologic, and clinical considerations | Kauke et al. | 2021 | Case report | 4 |
| DOI:10.3389/fneur.2020.593153 | Neuromotor Speech Recovery Across Different Behavioral Speech Modifications in Individuals Following Facial Transplantation | Eshghi et al. | 2021 | Cohort study | 3 |
| DOI: 10.1097/PRS.0000000000007890 | Face Transplant: Current Update and First Canadian Experience. | Govshievich et al. | 2021 | Case report | 4 |
| DOI: 10.1097/GOX.0000000000004038 | Facial Nerve Revascularization Strategies in Facial Restoration | Khajuria et al. | 2022 | Case series | 4 |
| DOI:10.1073/pnas.2211966120 | Re-cognizing the new self: The neurocognitive plasticity of self-processing following facial transplantation | Azevedo et al. | 2022 | Case report | 4 |
| DOI:10.1097/PRS.0000000000010242 | Facial Expression after Face Transplant: An International Face Transplant Cohort Comparison | Dorante et al. | 2023 | Cohort study | 3 |
| DOI:10.1002/ca.24247 | Anatomical study of trigeminal-facial nerve communications: Application to facial transplant surgery | Iwai et al. | 2025 | Cadaveric study | 5 |

**Supplemental digital content 3.** Quality assessment of studies included via the Newcastle-Ottawa Scale (NOS) scoring system

| **DOI** | **Study title** | **First author** | **Year of publication** | **Study type** | **Selection** | **Comparability** | **Outcome** | **NOS-score** |
| --- | --- | --- | --- | --- | --- | --- | --- | --- |
| DOI: 10.1097/PRS.0b013e3181954e8c | Mini-temporalis transfer as an adjunct procedure for smile restoration | Terzis et al. | 2009 | Cohort study | 3 | 1 | 2 | 5 |
| DOI: 10.1177/000348941312201106 | Long-term outcomes of facial nerve function in irradiated and nonirradiated nerve grafts | Leong et al. | 2013 | Cohort study | 3 | 1 | 2 | 5 |
| DOI:10.3389/fneur.2020.593153 | Neuromotor Speech Recovery Across Different Behavioral Speech Modifications in Individuals Following Facial Transplantation | Eshghi et al. | 2021 | Cohort study | 3 | 1 | 2 | 5 |
| DOI:10.1097/PRS.0000000000010242 | Facial Expression after Face Transplant: An International Face Transplant Cohort Comparison | Dorante et al. | 2023 | Cohort study | 3 | 1 | 2 | 5 |
